# AMLVaran: a software approach to implement variant analysis of targeted NGS sequencing data in an oncological care setting

**DOI:** 10.1186/s12920-020-0668-3

**Published:** 2020-02-04

**Authors:** Christian Wünsch, Henrik Banck, Carsten Müller-Tidow, Martin Dugas

**Affiliations:** 1 Institute for Medical Informatics, University of Münster, Albert-Schweitzer-Campus 1, Building A11, Münster, Germany; 2 Department of Medicine V, Hematology, Oncology and Rheumatology, University of Heidelberg, Heidelberg, Germany

**Keywords:** Genomics, NGS sequencing, Variant calling, Variant annotation, Variant filtering, Variant interpretation, Mutation database, Diagnostic report

## Abstract

**Background:**

Next-Generation Sequencing (NGS) enables large-scale and cost-effective sequencing of genetic samples in order to detect genetic variants. After successful use in research-oriented projects, NGS is now entering clinical practice. Consequently, variant analysis is increasingly important to facilitate a better understanding of disease entities and prognoses. Furthermore, variant calling allows to adapt and optimize specific treatments of individual patients, and thus is an integral part of personalized medicine.However, the analysis of NGS data typically requires a number of complex bioinformatics processing steps. A flexible and reliable software that combines the variant analysis process with a simple, user-friendly interface is therefore highly desirable, but still lacking.

**Results:**

With AMLVaran (AML *Var*iant *An*alyzer), we present a web-based software, that covers the complete variant analysis workflow of targeted NGS samples. The software provides a generic pipeline that allows free choice of variant calling tools and a flexible language (SSDL) for filtering variant lists. AMLVaran’s interactive website presents comprehensive annotation data and includes curated information on relevant hotspot regions and driver mutations. A concise clinical report with rule-based diagnostic recommendations is generated.An AMLVaran configuration with eight variant calling tools and a complex scoring scheme, based on the somatic variant calling pipeline *appreci8*, was used to analyze three datasets from AML and MDS studies with 402 samples in total. Maximum sensitivity and positive predictive values were 1.0 and 0.96, respectively. The tool’s usability was found to be satisfactory by medical professionals.

**Conclusion:**

Coverage analysis, reproducible variant filtering and software usability are important for clinical assessment of variants. AMLVaran performs reliable NGS variant analyses and generates reports fulfilling the requirements of a clinical setting. Due to its generic design, the software can easily be adapted for use with different targeted panels for other tumor entities, or even for whole-exome data. AMLVaran has been deployed to a public web server and is distributed with Docker scripts for local use.

## Background

Next-Generation Sequencing (NGS) enables large-scale and very cost-effective sequencing of genetic samples for the detection of mutations. Already being used in research worldwide, sequencing data is now starting to enter also routine care settings [1]. It can be a valuable instrument for a better understanding of the emergence and prognosis of a disease [2]. Also, it can help to optimize the type of treatment for personalized medicine [3].

Implementing NGS into clinical settings imposes a lot of challenges [4]. Most noticeably, analysis of raw sequencing data is complex due to the large variety of available bioinformatics pipelines and associated configuration parameters. With rapid development of new analysis pipelines that tend to produce highly differing result lists (“moving target”), bioinformatics expertise is required for an optimized analysis. Even sophisticated pipelines suffer from artifacts caused by immanent weaknesses of the sequencing technology. Discrimination between polymorphisms and pathogenic variants is not trivial, either. The type of tissue to be analyzed is important as well, since relevant variants tend to occur in low frequencies for tumor probes due to heterogeneous cell mixtures. The variety of available clinical databases complicates the annotation of identified variants, and their incompleteness leads to a high number of variants of unknown significance, calling for further (manual) inspection.

Clinical use requires software that is able to perform a complete bioinformatics analysis, while offering a user interface that can be operated by medical or laboratory personnel without a dedicated bioinformatics background. For clinical usage, it is furthermore critical to guarantee reproducibility and traceability of the generated results over long time periods. With ongoing research to clarify the clinical significance of unknown variants and increasing development of related systems in the clinical routine, software tools for the automated detection of disease-specific variants are increasingly relevant for routine clinical practice.

Acute Myeloid Leukemia (AML) is a myeloid disease that is particularly well characterized regarding genetic aberrations. The prognosis for affected patients is significantly influenced by certain driver mutations [5, 6]. With NGS-based techniques already being used in AML routine care, this leukemia entity is a particularly promising target for the design and evaluation of a flexible, automated and user-friendly bioinformatics software.

### Related work

Available solutions for variant analysis from NGS sequencing data include commercial products (e.g. the Qiagen CLC Workbench), which are closed-source and therefore provide only limited insights into variant calling and filter algorithms and only limited options for customization. Even more critical, commercial web-based tools (like VarSome [7]) typically require the sequencing data to be uploaded to the manufacturer’s servers. Open-source command line tools are available for a multitude of specialized subtasks of NGS analyses, but require more in-depth bioinformatics knowledge (e.g. BWA [8] for sequence alignment, GATK [9], FreeBayes [10], SamTools [11] for variant calling or SNPeff [12] or AnnoVar [13] for biological annotations, etc.). While McKerrel et al. present a complete pipeline optimized for AML specific source data [14], their software does not provide a graphical user interface, and therefore requires time-consuming configuration and adaptation by bioinformaticians. Furthermore, a number of generic graphical tools exist, which integrate different variant calling pipelines and aim to automate the variant calling and annotation process (e.g. Galaxy [15], Chipster [16], CoVaCS [17]). However, since the main focus of these tools is variant calling, their output is typically presented as basic lists of detected variants, which have to be further inspected and annotated to yield applicable information. Annotation tools like VariantDB [18] or VIS [19] start from a list of detected variants and can accumulate and manage annotation information from various sources, but are limited to basic research contexts. Neither of these tools addresses specific clinical requirements like reproducibility, long-term data storage or usability for non-bioinformaticians, and are therefore of limited use in a clinical setting. The combination of a complete variant calling pipeline, a management system for annotation databases and pre-curated literature data, detailed clinical reports and elaborated, interactive visualizations is desirable for use in routine patient care, but not primarily provided by any of these tools.

### Objectives

In this article, we present AMLVaran, the AML *Var*iant *An*alyzer. AMLVaran is a web-based software platform for analysis of somatic variants on targeted NGS data, addressing the requirements of a clinical setting. This platform shall cover the complete workflow from raw sequencing data to interactive clinical reports. It shall provide a flexible, modular analysis pipeline that can combine an arbitrary number of variant calling tools, and offer a generic model for variant filtering through a customizable scoring scheme. AMLVaran shall include a user-friendly interface that presents results in form of a structured clinical report with interactive features, which support further research. Furthermore, comprehensive curated data on therapy-relevant hotspot regions shall be incorporated, and presence, absence, or coverage of known driver mutations related to a chosen disease entity shall be provided. AMLVaran shall be tested for use with AML data, but is intended as a generic system adaptable to other cancer types. Since the software is designed for clinical application, AMLVaran’s focus is the generation of accurate and reliable results. We therefore apply the program to three AML datasets, and test its recall and precision to evaluate the software’s performance, 0.95 is targeted for recall and precision. Results of two user-studies shall provide evidence for AMLVaran’s practical usability by medical professionals.

## Implementation

### System architecture

AMLVaran is composed of three main components. First, a flexible, generic variant calling pipeline that generates variant lists from raw sequencing data. Second, an interactive website, which presents the analysis results together with interactive filter settings and creates a standardized, rule-based clinical report. A relational database storing both, results and annotation data, is the third integral part of AMLVaran.

The workflow consists of the following steps: A web-form manages the upload of one or more NGS raw sequencing files for analysis. Acceptable formats include aligned data in the Binary Alignment Map format (.bam, [20]) or raw data in fastq format [21]. The input files are subsequently processed by the variant calling pipeline, which invokes an adaptable number of variant calling tools and combines their results according to a customizable scheme. When the analysis is finished, the output can be inspected on AMLVaran’s web interface.

AMLVaran’s web interface provides four main functions for presenting its results, grouped into separate views. The first view lists basic information concerning the patient, and metadata for the sample that is currently being examined. The second one presents an overview of the mutation status for the predefined driver mutation sites. For each mutation site, additional information can be retrieved, such as manually curated information regarding the significance of the respective variant for the disease in question. A coverage plot and details about the detected mutations in the chosen region are integrated as well. Additionally, predefined diagnostic recommendations are included, which are derived from the mutation status of known driver mutation sites. The third view provides an interactive browser, which displays all detected variants. Results can be dynamically filtered and sorted by different criteria, e.g. variant allele frequency or read coverage. The last view features a genome browser, which allows to inspect the mutation of interest on the basis of aligned reads from the original sequence data.

The most important pieces of information from the dynamic website are composed into a static, clearly structured clinical report containing the diagnostic and therapeutically relevant details. A tracking system is provided, ensuring, that in case of updated annotation databases, the previously generated reports remain accessible. The web-interface is described in detail in the AMLVaran QuickStart Guide (see Additional file 2).

**Fig. 1:**
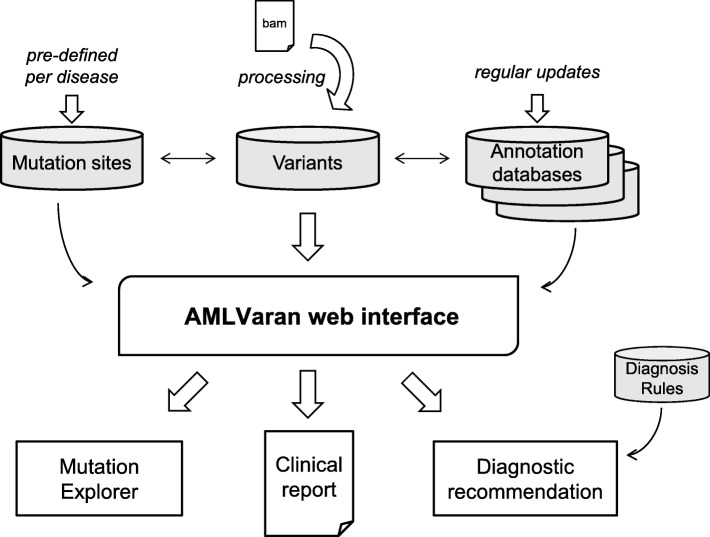
Overview of AMLVaran’s system architecture. Uploaded raw sequencing data are processed by the mutation calling pipeline and the resulting variants are stored in the database. For display at the web interface, the variants are compared with predefined mutation sites and combined with the most up-to-date information from annotation tables. Afterwards, the output is presented in form of a dynamic mutation explorer with comprehensive filter settings. A standardized clinical report is generated from the predefined driver mutation sites as well

The architecture of the main system components is shown in Fig. 1.

Data security aspects have been taken into account throughout development, details on which are provided in Additional file 1: Section 2.

### Implementation details

#### Generic variant calling pipeline

A central challenge for AMLVaran as well as related tools is the generation of reliable and clinically usable variant lists from a sequenced sample. Different variant calling tools generate highly differing variant lists [22], and several studies indicate, that none of the available open-source variant calling algorithms can obtain the sensitivity and specificity required for clinical usage [17, 23, 24].

It has been shown, that combining several variant calling tools can improve the quality of the resulting variant lists, but an optimized filtering strategy to remove false positive calls is equally important for satisfying results [25]. Chiara et al. [17] showed, that the use of three calling tools with a majority vote consensus strategy can lead to a valuable increase in sensitivity. By combining eight variant callers with a sophisticated score calculation strategy, Sandmann et al. achieved an even greater increase in sensitivity of up to 0.99 [24]. These improvements in detection of low frequency mutations, which are clinically relevant, in particular to detect minimal residual disease, now allow for reliable variant calling, especially with tumor samples.

Hence, we implemented a generic variant calling pipeline to support the combination of arbitrary variant calling tools. It was initialized with the tools used in CoVaCS [17] and appreci8 [24], but can be equipped with any other combination of variant callers as well. The results of the individual tools are then integrated via Variant Tools [26] and are subsequently filtered using a flexible, generic score calculation scheme.

**Fig. 2:**
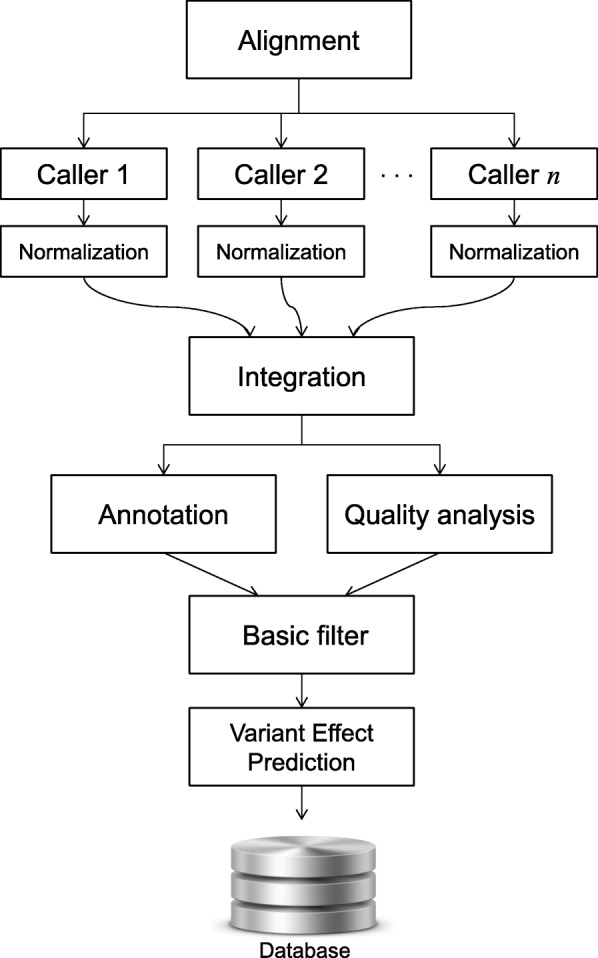
Flow chart of the generic variant calling pipeline featured in AMLVaran. An uploaded bam-file is processed by an arbitrary number of variant calling tools (configurable by templates). The resulting variant lists are then normalized and integrated into one common list (in vcf format). For the resulting list of variants, a functional annotation via SNPeff [12] and a calculation of sample-specific quality parameters (such as allelic frequency, base quality, etc.) from the bam file is performed via bam-readcount [27]. By means of a basic filter, low quality variants are excluded. For the remaining ones, an effect prediction using Provean [28] is carried out. The resulting data is stored in a database and then processed by an advanced filtering strategy

The workflow of the generic pipeline is shown in Fig. 2. A detailed documentation of the employed tools is provided in Additional file 3.

#### Storing variants

The Variant Call Format (vcf) [29] defines a standard for storage of genetic variants in a text file. However, the vcf definition allows equivalent variants to be represented in an ambiguous way. Even widely-used databases like dbSNP contain up to 14% non-normalized variants, and 4.6*%* unrecognized duplicates [30].

In order to get a unified, unique representation of all variants, a number of preprocessing steps is applied to AMLVaran’s variant lists, as well as the annotation databases. The preprocessing is described in Additional file 1: Section 1.

#### Variant filtering

The combination of variant callers increases sensitivity compared to only one tool, but at the expense of a higher false positive rate, if no additional filters are employed. Sandmann et al. [24] showed, that a simple consensus strategy as proposed before [17] cannot provide satisfactory results with regards to sensitivity and specificity. Sandmann et al.’s appreci8 therefore employs a more sophisticated strategy. The output from eight variant callers is combined, and thoroughly filtered with a complex score calculation system. An Artifact and Polymorphism score (A/P score) is calculated from various criteria [24], which can then be used to classify the variants. In principle, however, any rule-based filtering process of variant lists can be described by a criteria-based scoring scheme.

To further improve the filtering process, a formal language for the definition of variant filtering scores was developed and implemented. This language is flexible enough to implement complex scoring algorithms like appreci8, easily adaptable, and customizable by a graphical interface [31]. A scoring scheme is specified in JSON and consists of a list of criteria which contribute to the score by a defined weight. Individual criteria can be linked by logical operators AND and OR, and can include variable thresholds. The software is able to derive a graphical interface from the abstract scheme. This enables the end user to activate and deactivate individual criteria, and to adjust threshold values and weightings as needed.

**Fig. 3:**
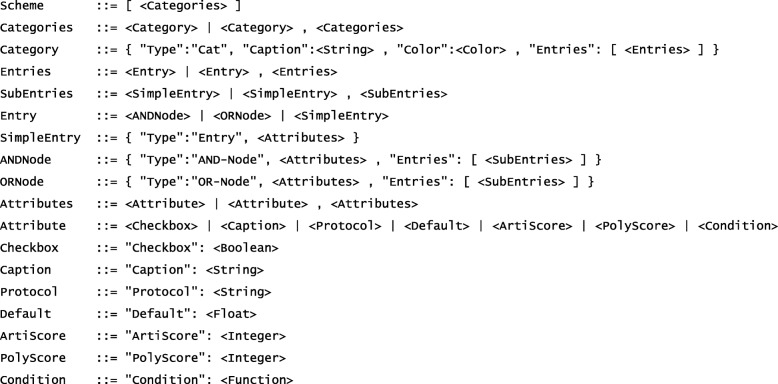
Formal definition of the SSDL in Backus-Naur-Form. A filter scheme consists of one or more categories, which can hold one or more entries, each of which is a rule that contributes to the artifact and/or polymorphism score. Entries can be combined by AND- or OR-nodes. Customizable thresholds can be included as well. The primitive data types (<String>, <Integer>, etc.) are defined canonically. <Function> is an external boolean function specifying the condition that has to be fulfilled for the rule to be applied. The function can utilize the syntax of the underlying R/JavaScript programming language. Access to the columns of the variant table is provided by the custom function “*x(columnName)*”. Other provided functions like “*isEmpty(string)*” or “*stringContains(haystack, needle)*” can be used in order to define language-independent filter schemes

The novel Scoring Scheme Definition Language (SSDL) is universally applicable in the sense that, once created, scoring schemes can be used not only within the AMLVaran platform, but also in standalone applications, websites, and in standard applications such as R or Microsoft Excel. Implementations in JavaScript, R, and Excel VBA are already publicly available [32]. A formal definition of the SSDL’s grammar in Backus-Naur-Form is provided in Fig. 3.

**Fig. 4:**
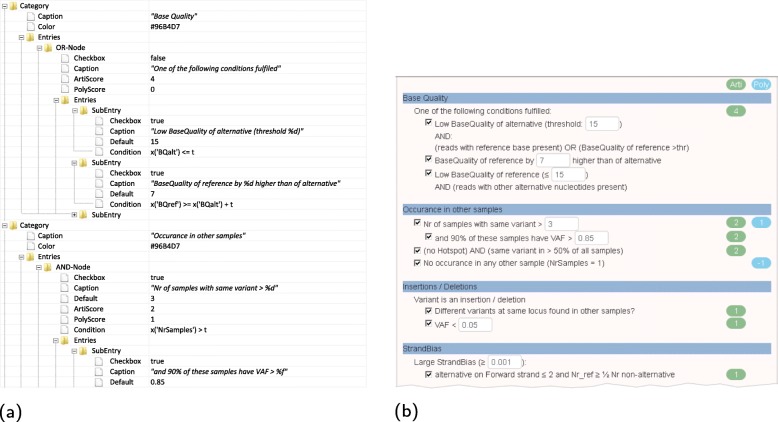
Generic model for A/P-score calculation. **a** Visualization of the generic, system-independent description in SSDL format. **b** The graphical panel for selecting and adapting the parameters for calculation of artifact and polymorphism scores is derived from the SSDL code shown in **a**. Elements such as categories, simple combinations like AND and OR, checkboxes and adjustable thresholds can be freely combined. The thresholds can be adapted, and the score calculation strategy can be fully customized. By default, a score calculation scheme based on appreci8’s complex filtering strategy is provided (see Additional file 4)

A subset of the features of SSDL, as well as the resulting graphical user interface, is shown in Fig. 4.

Furthermore, a default scoring scheme for the appreci8 filtering process is available from our website[33] and from the AMLVaran package.

#### Gathering annotation data

For adequate biological and clinical interpretation of variants, annotation with comprehensive and up-to-date biological information is a necessity. However, clinical annotation databases are often updated on a regular basis, and there are several options to address these changes [34].

On the one hand, there is a strong medical motivation to always consider the most recent biological data regarding the detected mutations for both old and new samples. On the other hand, it is required to reproduce previous reports based on annotation from the past, for practical as well as legal reasons.

To meet these requirements, an interactive online report equipped with the latest available biological annotations is presented to the user. Simultaneously, an archive of all previously generated reports is stored on AMLVaran’s server. A report is automatically saved in PDF format each time the results page is accessed. As proposed by Cutting et al. [34], references to a set of online annotation sources are integrated, allowing for easy access to most recent annotation sources.

The technical implementation features stored variant lists and annotation data in separate tables within the database. Whenever a sample is retrieved, relevant data are merged.

Currently, AMLVaran uses dbSNP [35], COSMIC^1^ [36], ClinVar [37], dbNSFP [38], 1000Genomes [39], ESP6500 [40], ExAC [41], as well as functional prediction data from PROVEAN [28] and SIFT [42] for variant annotation.

#### Identifying diagnostically relevant mutations

For certain cancer types such as AML, specific driver mutations are known to influence the prognosis of the patient, as well as the choice of therapy. Several of these are summarized in the WHO Guidelines of 2017 [43] for AML. Precise mapping of the detected mutations to the listed reference mutations is necessary for an automated application of these guidelines. This mapping process is aggravated by differing levels of accuracy regarding mutation positions in the literature. While a point mutation in DNMT3A at position R882 that is associated with a bad prognosis in AML [44] is well defined, a 4 bp frameshift insertion in NPM1 *around* position 960 (chr5:170837544) has rather positive effect on therapy response [45], however its actual position can vary. Similarly, c-KIT insertions/deletions anywhere in exon 8 are associated with an increased risk of relapse [46].

For the hotspot overview, a clear, unambiguous and reproducible identification of a mutation site is therefore needed, regardless of the different kinds of mutation descriptions. To achieve this, all mutation sites are stored in AMLVaran’s databases with precise genomic coordinates. As set of genomic ranges (from 1 bp to complete exons) can be associated with each mutation. Additionally, restrictions to certain mutation types like frameshift insertions can be specified. Any matching, non-synonymous, not benign variant entry located in the specified areas is then regarded as driver mutation.

However, it is equally important to reliably rule out the presence of relevant driver mutations. This requires, that no relevant mutation was found in the corresponding region, but also that the coverage of the entire region is sufficiently high. Only then a site is classified as “wild type” by AMLVaran.

Implications defined in any disease specific treatment guidelines can be automatically derived on the basis of the detection or exclusion of these defined driver mutations, and are displayed as diagnostic recommendations. The rule-based system is stored within the database, and can be edited by the system’s administrator.

#### Clinical reports

**Fig. 5:**
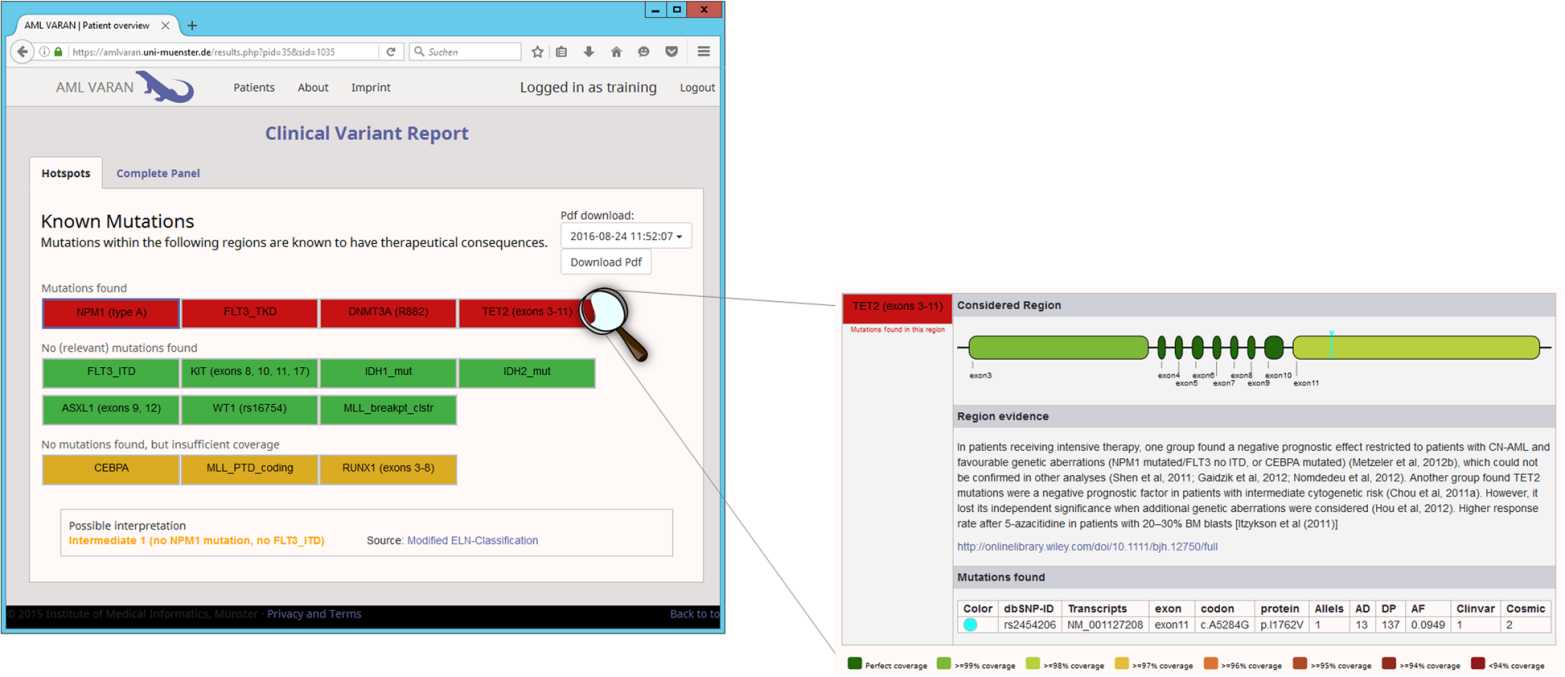
Visualization of a sample clinical report. AMLVaran’s clinical reports consist of an overview of the predefined mutation sites. For each site, a color-coded area shows one of three states. Red indicates that a relevant mutation was found in the given area, yellow intervals have no associated mutation, but the coverage is not sufficient to allow a safe exclusion of variants. Finally, the presence of mutations in green areas can reliably be excluded due to good coverage, and lack of mutation evidence. Based on the mutation status and the stored diagnostic rules, the applicable diagnostic information is displayed. Furthermore, an assessment summary can be added by the evaluating pathologist. The report can be augmented with more detailed information for each mutation site, including details on the detected mutations, the coverage in the examined area, and general information on the function of the considered gene

In interviews with clinical users, a need for a quick and standardized way of transferring AMLVaran’s results into electronic healthcare or archiving systems was expressed. To fulfill this demand, a standardized PDF report as shown in Fig. 5) is created for each sample, containing the most relevant information for clinical diagnostics. In particular, it includes the detected or excluded driver mutations, the coverage distribution in the stored hotspot areas, as well as predefined curated information about clinical implications with references to corresponding studies. These reports are structured from coarse to fine [34], starting with an overview of the predefined hotspot mutation sites and their status, as well as displaying more detailed information on each of the hotspot areas. In order to ensure reproducibility of medical results, further details are provided, e.g. type and version of the analysis.

#### Interactive variant explorer

**Fig. 6:**
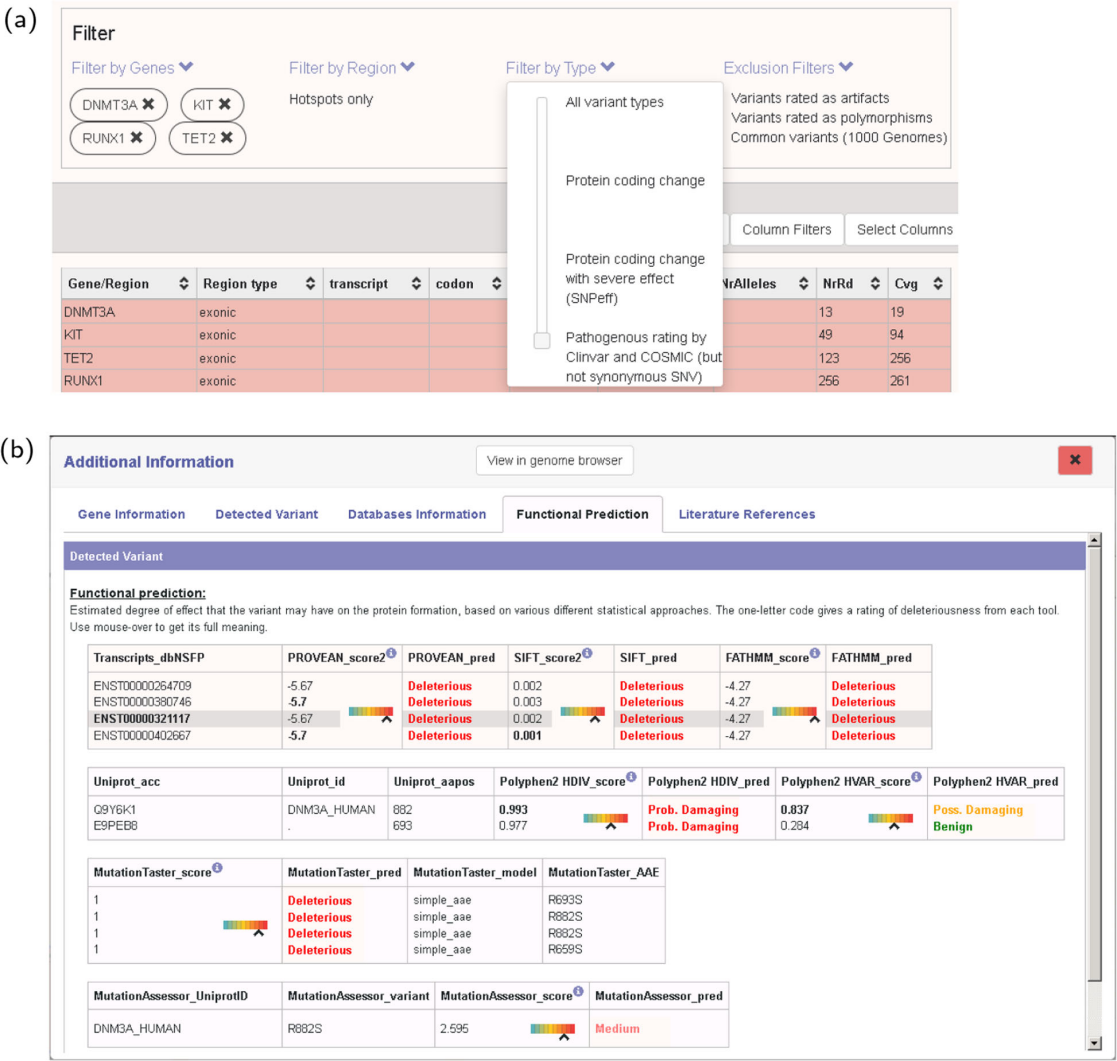
Overview of AMLVaran’s variant explorer. **a** Main view of the variant explorer. The detected variants are presented as a dynamic, customizable, interactive table that allows for comprehensive, easy-to-use real-time filtering. Variants can be filtered by gene name in the displayed gene panel, which shows all genes with their number of variants and allows the user to restrict the output to one or more genes. Further filtering options include the region type (exon, intron, hotspot regions), mutation type (non-synonymous, protein-coding, pathogenous effect prediction), and filtering by minimum quality values (coverage, variant allelic frequency, base quality). Certain kinds of variants can be excluded, e.g. variants rated as artifacts or polymorphisms by appreci8. The presented table can be easily adjusted by selecting the columns to be displayed, changing the sorting order and adding custom filters per column. **b** Detailed view of all annotation information available in AMLVaran for a variant. Information is divided into different tabs, which show the affected gene (name, function, pathways), the detected variant (protein effect, allele frequency, base quality), the effects of the variant in various medical databases, functional prediction scores from eight tools including rank scores with graphical representation, and a large number of literature references to the chosen gene and variant, retrieved on-the-fly from the constantly updated CiVIC database [49]

In addition to already known clinically relevant driver mutations, assessment of further mutations can be relevant for certain cancer types [47]. Clinical users expressed interest in comprehensive and up-to-date information on these variants, and for interactive overviews in order to enable quick and accurate interpretation. While AMLVaran retrieves known clinical information from annotation databases like ClinVar or COSMIC, additional resources are required for those mutations. Hence, functional prediction tools, conservation scores and mutation frequency in sequencing projects, such as 1000 genomes, were integrated into the platform, and provide information on the potential pathogenicity of variants. The AMLVaran software integrates available information for each detected variant within an interactive variant explorer, together with adjustable filtering and sorting options (Fig. 6a). All outputs of the variant explorer, with applied scoring, filtering and sorting settings, can be exported for further processing in tabular form.

For each variant, a detailed view with all available annotation can be selected, including eight functional prediction scores. For each functional prediction tool, the Converted Rank Score from dbNSFP [48] is displayed to support assessment of harmfulness of the current mutation, compared to predictions of all other non-synonymous coding SNVs (Fig. 6b).

In addition, it is possible to inspect aligned reads for each mutation site with the integrated genome browser (IGV [50]).

## Results

AMLVaran was installed on a virtual server with high performance hardware, and on a standard desktop workstation. Detailed information regarding hardware configuration and runtime is provided in Additional file 1: Section 5. The software’s performance was evaluated on AML and MDS datasets, and tested with regards to its sensitivity, positive predictive value, and general usability.

Three datasets were analyzed with the generic pipeline in its default appreci8-based configuration, with the following eight variant calling tools: GATK 3.3^2^ [9], FreeBayes 1.0.2 [10], SamTools 1.3 [11], LoFreq 2.1.2 [51], Platypus 0.8.1 [52], SNVer 0.5.3 [53], VarScan 2.4.0 [54], and VarDict (Java) 1.5.5 [55].

For annotation and variant filtering process, the following databases were used: dbSNP v150 (2017-04-03) [35], ClinVar (2017-12-31) [37], COSMIC v83^3^ (2017-11-07) [36], 1000Genomes v5b (all, Aug 2015) [39], ExAC 65000 v0.3 (all, 2015-11-29) [41], and ESP6500 (2014-12-22) [40].

Precalculated prediction scores were downloaded from PROVEAN v1.1 [28].

The test system with results on the described datasets are available at https://amlvaran.uni-muenster.de[56].

### Validation of the generic variant calling pipeline

#### Validation strategy and source data

To validate the generic AMLVaran pipeline in its appreci8-like configuration, its output was compared to the original appreci8’s output.

AMLVaran was used to analyze a set of three published datasets. Two of them were well characterized datasets from myelo-dysplastic diseases, for which biological validation data was available (MDS-1 and MDS-2). The third dataset (AML-1) was taken from routine AML diagnostics, which resembled the intended use case, but only limited validation data was available.

MDS-1 and MDS-2 were collected and sequenced within the European MDS Triage project and focused on patients with MDS. The latter one was provided by the University Hospital of Halle, and contained a mixed set of samples (67x AML, 1x AL, 2x t-AML, 2x s-AML after MDS, 1x MDS/AML unsure diagnosis, 17x MDS, 19x MPN and 10x unknown diagnosis).

Source data is available for download from the NCBI Sequence Read Archive (BioProjectID: PRJNA388411 [57]). The raw variant result tables are provided in Additional files 5, 6, 7 and 8.

#### Evaluation on hotspot regions

For clinical use, it is crucial, that existing variants are detected with very high reliability. No less important, however, is the pipeline’s ability to safely exclude the presence of mutations for a chosen position. This is of special importance for hotspot regions, which are typically designed to contain mutations relevant for treatment.

First, we evaluated results within these defined, therapy-relevant areas for all three datasets. For both MDS and AML datasets, there was a high overlap between the two pipelines and the biological truth. In total, 415 out of 419 variant calls matched for AMLVaran and appreci8 and the ground truth, leading to sensitivity ≥0.99, and PPV ≥0.99. The only two cases of discrepancy between AMLVaran and appreci8 were borderline cases that were reported by only two or three out of eight variant callers, respectively. Differences between dbSNP versions changed the ratings of those variants in AMLVaran’s output.

A detailed comparison of the detected variants within these regions is provided in Table 1.

**Table 1:** Accumulated numbers of variants in hotspot regions detected by AMLVaran in comparison with appreci8 reference implementation

	MDS-1 (n=237, #Mut=195)		MDS-2 (n=46, #Mut=52)		AML-1 (n=119, #Mut=172)	
	appreci8	AMLVaran	appreci8	AMLVaran	appreci8	AMLVaran
Called variants	193	194	52	52	172	171
True positives	193	193	52	52	172	171
False positives	0	1	0	0	0	0
Sensitivity	0.9897	0.9897	1.0000	1.0000	1.0000	0.9942
PPV	1.0000	0.9948	1.0000	1.0000	1.0000	1.0000

#### Evaluation on the complete target region

Next, we considered the whole sequenced target region of 42 kbp length, which includes segments of 19 genes. The target region’s average coverage was 1450.

Almost all true positive calls from appreci8’s reference implementation were confirmed by AMLVaran. For dataset MDS-1, 395 of 397 true variants were identified, and all 89 variants of the MDS-2 dataset overlapped in both outputs. Likewise, 16 of appreci8’s 17 false positive calls for dataset MDS-1 and all three false positive calls for dataset MDS-2 were matching in AMLVaran’s results.

The observed differences can be explained by an improved normalization procedure and updated reference databases. Due to these improvements, the sensitivity of AMLVaran’s pipeline (0.98) was higher than of the reference implementation (0.95) for MDS-1. For MDS-1, 12 previously filtered true positive variants were correctly classified by AMLVaran, and another six new high-quality candidate variants were identified, for which validation is pending.

In general, AMLVaran’s pipeline was slightly more sensitive than the original appreci8 implementation, resulting in 12 additional false positive calls that were reported by AMLVaran. These differences were caused by changes in the updated dbSNP version (10 variants), and a corrected bug in appreci8’s method for the counting of samples (2x).

For dataset MDS-2, AMLVaran recognized all 89 validated mutations. Another promising candidate mutation as well as five additional false positive calls were reported. Reasons for the differences were differing PM flag due to newer dbSNP version (three variants), an update to the integrated COSMIC version (2x), and improved InDel normalization (1x).

**Fig. 7:**
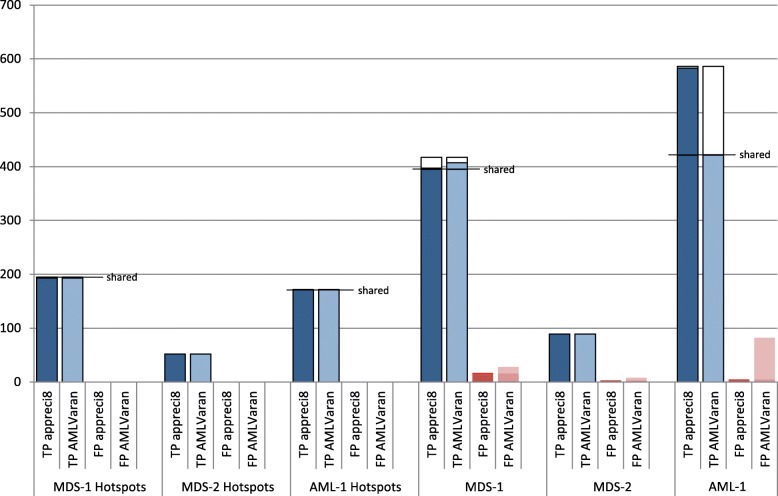
Classification of variant calls per dataset. The counts for true positive and false positive detections are shown for all three test datasets. For all subplots, a black outline shows the variants validated as true positives (ground truth). Of these, the number of detected true positives for the appreci8 reference and AMLVaran’s results are visualized as blue bars. A horizontal line indicates the number of variants that overlap in both pipelines. Further to the right side, the number of false positive calls from appreci8 and AMLVaran is displayed in red. False positives reported concordantly by both pipelines are indicated by a darker shade of color. **a** MDS-1: *n*=237, #Mut=417, #TruePos=407, #FalsePos=28, Sens=0.9760, PPV=0.9563. **b** MDS-2: *n*=46, #Mut =89, #TruePos =89, #FalsePos =8, Sens =1.0000, PPV =0.9175. **c** AML-1: *n*=119, #Mut =586*, #TruePos =422 (*) no biological validation data available

In total, AMLVaran’s variant lists showed high concordance with the reference results for both MDS datasets. A detailed comparison of the results of both pipelines and the biological ground truth is shown in Fig. 7.

#### Evaluation of dataset AML-1

For dataset AML-1, the investigated target regions were much larger. In total, 523 kbp in 112 genes were analyzed, with an average coverage of 570 and over 90% of the unfiltered calls were artifacts/polymorphisms. Since this dataset originated from routine diagnostics, biological validation data was limited to a part of the samples and to the genes NPM1 (*n*=75), JAK2 (*n*=10), FLT3_TKD (*n*=50), and CEBPA (*n*=30).

For these biologically validated sites, AMLVaran detected 51 out of 55 variants. In addition, AMLVaran did not report a single false positive call for any of the 110 validated wild-type alleles. While the program did report two unexpected calls for FLT3-TKD, these two variants were also reported by appreci8 and validated by expert inspection afterwards.

**Fig. 8:**
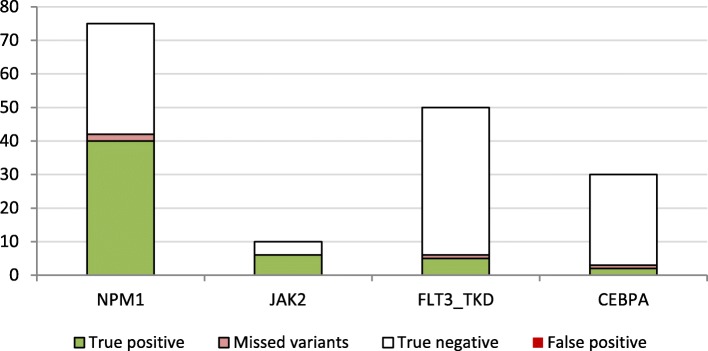
Results for validated mutations in the AML-1 dataset. The graphic shows the total number of validated samples per variant as a black outline. The number of true positive detections by AMLVaran is visualized in green, and the number of validated true variants that were missed by the program are indicated in pale red. The remaining white part of the bar charts indicate positions without calls in AMLVaran, which were confirmed to be wild-type alleles. There were no false positive detections. **a** NPM1: *n*=75, Detected =40/42, Sensitivity =0.9524, PPV =1.0000. **b** JAK2: *n*=10, Detected =6/6, Sensitivity =1.0000, PPV =1.0000. **c** FLT3_TKD: *n*=50, Detected =5/6, Sensitivity =0.8333, PPV =1.0000. **d** CEBPA: *n*=30, Detected =2/3, Sensitivity =0.6667, PPV =1.0000

Compared to the biological truth, AMLVaran achieved a high number of overlapping variants, resulting in high sensitivities of 0.95 resp. 1.00 for the NPM1 and JAK2 variants. For FLT3-TKD, one variant was missed. A sensitivity of 0.67 or higher was achieved for CEBPA, a gene that is typically correlated to coverage issues and high numbers of false positive and false negative variants [58]. Detailed results for all groups of validated mutations are shown in Fig. 8.

In total, AMLVaran reported 504 variants in 119 samples for dataset AML-1, ranging from 0 to 13 variants per sample, and with an average of 4.2 calls per sample. In contrast to datasets MDS-1 and MDS-2, more differences between AMLVaran and appreci8 were noticeable. A set of 426 variants matched between AMLVaran and appreci8, however 78 variants were called by appreci8 only, and 162 additional variants were specific for AMLVaran. It stands to reason, that these differences are caused by the same effects that were discovered for datasets MDS-1 and MDS-2 (database updates and improvements to normalization technique). Results for validated variants indicated that deviations preferentially affected cases of high ambiguity. Hotspot mutations with high coverage or other unambiguous variants were rarely affected.

### Evaluation of the system’s usability

The development of AMLVaran was driven by a focus group, consisting of computer scientists as well as several clinical domain experts and medical professionals. The involvement of clinical experts was especially valuable to identify and prioritize user requirements. Two user studies were conducted during the development process to ensure that the software met critical requirements and offered good usability. In the first user study, a prototype of the software [59] was evaluated, while the final AMLVaran program was examined in the second study. 9 and 14 independent medical professionals were asked to fulfill application-related tasks using AMLVaran, respectively. Afterwards, participants were asked to rate their experience with regards to the software’s general performance, functionality, and user-friendliness.

During the first test of the prototype, all 9 users had difficulties with the interpretation of the presence or severity of given hotspot mutations. On average only 4.3 of 8 test cases were solved correctly. None of the participants was able to answer all 8 cases correctly. Three persons achieved 6 of 8 points, and three persons 3 points. From these test results and unstructured interviews with the test candidates, six major usability issues were identified, which are described in Additional file 1: Section 3.

Detailed feedback from the study was used to address the mentioned key factors and to optimize a number of features during the continued development of the software. Data presentation was improved by a new, clear structuring of related views. A detailed view for each variant was composed and grouped by categories (Fig. 6b). The use of more intuitive headings, tool-tips, additional explanatory texts and improved predefined filtering options helped to increase the interpretability of AMLVaran’s results. A major improvement was the introduction of the central overview of the mutation status for known driver mutations (Fig. 5). In this overview, both detection and exclusion of these mutations were represented as intuitive traffic light color codes.

After these improvements, a second user study was carried out with a different user group of 14 experts, and updated tasks. Regarding the interpretation of hotspot mutations, 5 out of 6 test cases were now correctly interpreted by all 14 participants. The sixth test case was excluded due to ambiguity of the data. In addition to the improved performance, the second group of users also reported a higher degree of confidence while using the software during the interviews.

Finally, the perceived usability of the program was rated by participants of both studies with the help of a standardized questionnaire (System Usability Scale [60]), which provides a score between 0 and 100 (100 = best usability). AMLVaran’s prototype achieved an average score of 69 (median: 67, interquartile range: 14). The final version of the software reached an average score of 74 (median: 80, IQR: 28), which indicated a notable increase in user-friendliness. For the second study, 2 out of 14 test persons had to be excluded from the SUS rating, because they did not fully complete the test.

**Fig. 9:**
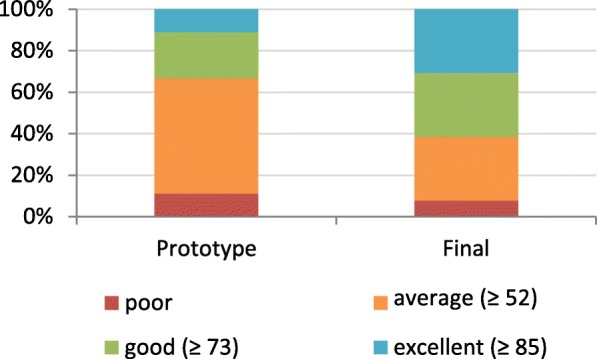
Rating of AMLVaran’s usability (SUS) by test persons. The graphic shows the comparison of the prototype’s performance with the final version of the software. The bars indicate the percentage of users who rated the software with a SUS score corresponding to categories “excellent”, “good”, “average” or “poor”. The final version was rated with “good” or better by 62% of the participants, while only 33% of the prototype users gave this judgement. A full third of the users considered the final system to be “excellent”. For both user studies, one user rated the system as “poor”

According to a study of Bangor et al. [61], scores between 60 and 80 are typical results for average software systems. Systems with scores of 73 or above are considered as “good”, 85 or greater signals “excellent” usability. It should be noted, that users received no training before carrying out the study. Figure 9 shows the distribution of scores assigned by the participants of both user studies.

While the increase of the usability score was only moderate, the overall complexity of the software and the difficulty of tasks increased in the final version. Since these tasks required a higher degree of coordination from the users, the results indicate, that the second system version was more usable.

## Discussion

High sensitivity and precision of variant calling are basic, but critical requirements for a successful application to clinical diagnostics. In the presented setting, sensitivity ≥0.98 and PPV ≥0.92 (≥0.99 for hotspot regions) were reached for validated datasets, which should be generally sufficient for clinical use. However, the signal quality and the incidence of mutations per gene needs to be considered for a thorough interpretation.

Considerable differences between AMLVaran and the appreci8 reference implementation were limited to dataset AML-1, and were largely caused by an improved normalization method and different versions of annotation databases. For updated versions of appreci8, we therefore expect smaller differences between this pipeline and AMLVaran.

Normalization of the different callers’ variant lists before combination, as well as the use of newer annotation databases, had a relevant influence on the outcome of the filtering process. In particular, the PM flag in the dbSNP database, which plays a crucial role in the appreci8 filtering scheme, is subjected to frequent changes, making the pipeline sensitive to database updates. Although biological validation was lacking for the AML-1 dataset, we assume, that the pipeline optimizations led to improved precision in comparison to the original appreci8 output due to the apparent improvements in the newer dbSNP version.

The deviations on dataset AML-1 show, that the output of individual callers and different pipelines can differ greatly, even for low-volume targeted sequencing data. We demonstrated, that existing variant calling pipelines are very sensitive to small adjustments of analysis parameters, an effect that is most notable in regions with lower data quality. It is therefore important, that these parameters are chosen based on a sufficient amount of valida- tion data for each dataset to account for differences in sequencing depth, differences in sample preparation, and other parameters of data generation. This is especially important for the design of a complete variant calling pipeline, which combines several variant calling tools to achieve optimal results.

In general, the performance of the presented pipeline can be improved through the incorporation of additional information regarding disease type and hotspot data. AMLVaran’s default appreci8-like analysis pipeline takes such information into account. In our datasets, the sensitivity and precision of variant calling was higher for hotspots than on the whole target region. Even with dataset AML-1, 171 of 172 calls in hotspot areas were matching between both pipelines. This might be explained by the fact, that some regions of the genome are difficult to sequence in general and therefore the knowledge about these regions has limitations.

For clinical use, not all detectable mutations are of the same relevance. In this scenario, it is often more important to identify or exclude therapy-relevant mutations than finding variants of unknown significance. Whole-genome sequencing with lower coverage and without special consideration of hotspots is therefore expected to yield data that is less suited for highly sensitive variant calling in regions of therapeutic interest. Consequently, at present, targeted sequencing appears to be the best choice for clinical diagnostics.

### Strengths

To the best of our knowledge, AMLVaran is the first freely available web-based software that combines a sophisticated but generic variant calling pipeline, a complex filter strategy, and disease-specific evaluation of predefined mutations in a form that is suitable for clinical application.

The novel Scoring Scheme Definition Language (SSDL) allows a standardized, reproducible, cross-platform definition of complex variant filtering systems.

Both, sensitivity and precision of the variant calling reach the specified goal of 0.95 or higher for hotspot regions in all three evaluated datasets.

The software not only supports confirmation, but also exclusion of predefined driver mutations by means of an integrated coverage analysis. This aspect is often lacking in common variant calling pipelines.

Since personalized medicine and genetic diagnostics is a moving target, frequent changes and updates of medical knowledge have to be taken into account to guarantee optimal variant filtering and interpretation. AMLVaran stores variants and annotation data separately. This flexible annotation approach enables to add, update or remove complete annotation databases at any time. There is no need to explicitly re-annotate previously analyzed samples after a database update.

The databases stored on AMLVaran’s server can also be used for independent real-time annotation without a full AMLVaran analysis [32].

In addition to the functionality, usability of a software system is a key success factor with regards to its practical use. We therefore assessed the usability of AMLVaran with medical experts, and refined the software iteratively. The proportion of users which rated the software with “good” or better increased from 33% to 62%. This clearly demonstrates the need for usability studies in clinical NGS software.

It stands to reason, whether a probabilistic approach of variant prediction could be employed instead of binary classification, in order to further improve prediction quality, especially for borderline variants. A detailed discussion of this idea is included in Additional file 1: Section 4.

### Limitations

The distinction between somatic mutations and artifacts or polymorphisms is a complex challenge, especially for tumor samples with potential subclones and lower expected variant allele frequencies. We chose a combination approach of several variant callers and demonstrated, that AMLVaran’s results had a high overlap with the reference pipeline’s variant lists. However, a productive implementation of AMLVaran into a clinical routine setting requires further validation through established sequencing methods (e.g. Sanger sequencing [62]).

Currently, AMLVaran can detect only SNVs and small InDels at the required precision rate for clinical use. Gene fusions or larger tandem duplications, such as FLT3-ITD, are much more difficult to detect by targeted data. However, McKerrel et al. have shown, that it is technically possible to detect structural variants from targeted NGS data, as long as a suitable sequencing panel is used [14]. With appropriate data and choice of precise detection tools, AMLVaran can be extended to detect those variants as well.

AMLVaran has been designed with a clear focus on analyzing single samples for certain diagnostically relevant mutations. Family-based analyses or investigation of new mutations in a cohort of samples are not in focus of this tool. The variant calling algorithms have been optimized for and tested with tumor data only, which requires special care due to mixture of cell material and high intra-tumor heterogeneity. Especially, somatic mutations may be observed at arbitrary low frequencies in tumor probes [63–65]. This is, why a highly optimized cancer-pipeline (appreci8) has been selected for AMLVaran, that utilizes eight callers to provide best sensitivity at the cost of a higher runtime.

AMLVaran might be applied for germline studies as well, but the variant calling and filtering algorithms should be adapted for this purpose.

### Transferability / universality

AMLVaran is a generic application for NGS variant analysis, as it offers a freely customizable scoring scheme interface, which can be used to define a multitude of score calculation and filter schemes. Furthermore, it can be equipped with any variant calling tools, and different targeted sequencing panels are available for selection. Custom panels can be imported via the web interface as well.

To adapt AMLVaran for other diseases, predefined driver mutation sites have to be changed and the rule system for treatment guidelines is to be adapted in the database.

Although AMLVaran has been developed and optimized for targeted NGS sequencing, it may be used for whole-exome (WES) data as well. Some notes on recommended adjustments, as well as a comparison of runtimes and number of detections when used with a WES germline sample can be found in Additional file 1: Section 6.

### Future work

In a pre-clinical user study, AMLVaran was well accepted by medical experts. The next step towards clinical use would be prospective evaluation and validation in a suitable clinical setting. At a later stage, integration in the Electronic Health Record system would be advantageous.

## Conclusions

With AMLVaran, we have developed a software system that enables variant analysis on NGS sequencing data and fulfills central requirements for the utilization in clinical routine care.

The presented software platform performs highly reliable variant calling, and achieves sensitivities of at least 0.95 on several datasets, as well as precision scores of at least 0.92, with a minimum precision of 0.99 on hotspot regions. None of the eight variant callers was able to achieve such high values on the tested datasets, when applied alone. This underlines the need for validation of present NGS pipelines before use.

AMLVaran is flexible enough to employ and combine arbitrary variant calling tools, and provides a user-friendly interface that has been evaluated in order to facilitate use by medical staff. The system’s performance is sufficient to perform analyses on standard desktop personal computers within less than one hour for targeted data, and within 24 hours for WES data.

From our perspective, the approach of AMLVaran can be a valuable contribution for use of NGS data in a clinical context. We demonstrated that fine-tuning of NGS pipelines with reproducible filtering of NGS results and usability analysis is needed to provide valid variant calling results which are comprehensible for medical users.

## Availability and requirements

**Project Name:** AMLVaran**Project home page:**https://amlvaran.uni-muenster.de [56]**Source code repository:**https://github.com/cwuensch/AMLVaran [66]**Operating system(s):** Linux**Programming language:** PHP, JavaScript, Python**Other requirements:** Apache web server, MySQL, Java 1.8, Python 2.7, VariantTools 2.7.0, samtools 1.3, vcftools 0.1.15, SNPeff 4.2, bam-readcount 0.8.0, reference genomes and the variant calling tools**License:** GNU GPL v3**Any restrictions to use by non-academics:** noneThe presented web platform AMLVaran is available for demonstration purposes at https://amlvaran.uni-muenster.de[56].

The following credentials can be used to view the published test datasets:

MDS-1: user: “test1”, password: “Sweden2017”.

MDS-2: user: “test2”, password: “Sweden2017”.

AML-1: user: “test3”, password: “Halle2015”.

(Upload and analysis of own samples is purely for research purposes and only possible after contact with the corresponding author.)

The source code is freely available from GitHub at https://github.com/cwuensch/AMLVaran [66], and can be adapted for new projects under the provided license.

A Docker installation script is available that guides a bioinformatician through the process of assembling and configuration of the software components.

A QuickStart Guide that helps with setup and usage of the software is provided in Additional file 2.

**Notice:** This is open source test code. The software components need to be adapted to local requirements. Especially the variant calling parameters are to be adapted for the type of data to be used. Importantly, the system needs to be assembled and validated locally before use! This code is provided “AS IS” and any express or implied warranties, including, but not limited to, the implied warranties of merchantability and fitness for a particular purpose are disclaimed.

**Datasets:** The datasets used for validation of the software are available for download from the NCBI Sequence Read Archive (BioProjectID: PRJNA388411; https://www.ncbi.nlm.nih.gov/bioproject/PRJNA388411[57]).

## Supplementary information

**Additional file 1** A PDF document with additional detailed information.

**Additional file 2** A PDF document, giving basic installation and usage instructions for the software.

**Additional file 3** The variant calling tools and parameters, as well as filter scheme used to generate the described results.

**Additional file 4** AMLVaran’s default variant scoring and filter scheme which resembles the appreci8 algorithm.

**Additional file 5** Variant calling results from dataset MDS-1 (MS Excel)

**Additional file 6** Variant calling results from dataset MDS-2 (MS Excel)

**Additional file 7** Variant calling results from dataset AML-1 (MS Excel)

**Additional file 8** Variants and validation for dataset AML-1 (MS Excel)

## Data Availability

The datasets supporting the conclusions of this article are available from the NCBI Sequence Read Archive (BioProjectID: 388411 [57]).

## Abbreviations

#Mut: Number of mutations
#TruePos: Number of true positive calls
A/P-score: Artifact / Polymorphism score
AF: Allele frequency
AL: Acute leukemia
AML: Acute myeloid leukemia
AMLVaran: AML variant analyzer
bam: Binary aligned map
bed: Browser extensible data (a list of genomic regions)
bp: base pairs
CPU: Central processing unit
DNA: Deoxyribonucleic acid
e.g.: exempli gratia - for example
EHR: Electronic health record
GB: Gigabyte
GHz: Gigahertz
InDel: Insertion / Deletion
IQR: Interquartile range
JSON: JavaScript object notation
kbp: kilo base pairs
MDS: Myelo-dysplastic syndrome
MPN: Myeloproliferative neoplasm
NGS: Next-Generation sequencing
PDF: Portable document format
PM: PubMed
PPV: Positive predictive value
RAM: Random access memory
resp: respective
SNP: Single nucleotide polymorphism
SNV: Single nucleotide variant
SUS: System usability scale
SSDL: Scoring scheme definition language
UTR: Untranslated region
VBA: Visual basic for applications
vcf: Variant call format
WES: Whole-exome sequencing
WGS: Whole-genome sequencing
WHO: World Health Organization

## Footnotes

1 COSMIC is not available for public usage due to license restrictions.

2 GATK is only available for public usage from version 4 and higher due to license restrictions.

3 COSMIC is not available for public usage due to license restrictions by the authors.
