# Supplementary material for: AMLVaran: a software approach to implement variant analysis of targeted NGS sequencing data in an oncological care setting
Source: BMC Med Genomics. 2020 Feb 4;13:17. doi: 10.1186/s12920-020-0668-3 (PMC7001226; doi:10.1186/s12920-020-0668-3)
Supplement: Supplementary file 1 — Additional file 1 A PDF document with additional detailed information. [file 12920_2020_668_MOESM1_ESM.pdf]

Supplement

1 Normalization and preprocessing of variant lists

The Variant Call Format (vcf) [1] defines a standard for storage of genetic variants in a text file. However, the vcf definition allows equivalent variants to be represented in an ambiguous way. For example, in the gene NPM1's transcript NM_002520.6, four valid terms encode the same frameshift insertion: *c.860_863dup*, *c.859_860insTCTG*, *c.861_863insTGTC* and *c.863_864insTCTG* (chr5:170837543 G↦GTCTG, chr5:170837545 C↦CTGTC, chr5:170837547 G↦GTCTG). During the integration of variant lists from different variant callers, this ambiguous representation can lead to multiple, but equivalent representations for the same variant and failing associations. It has been shown, that even well-known databases like dbSNP contain up to 14% non-normalized variants, and 4.6% unrecognized duplicates [2].

In order to get a unified, unique representation of all variants, a set of preprocessing steps is applied to AMLVaran's variant lists.

(1) Multi-allelic variants are split into single vcf entries for every occurring alternative allele. Allele-specific fields like the allelic frequency are separated accordingly.

(2) Multi-nucleic variants are separated into single nucleotide variant (SNV) entries for any nucleotide that has been modified.

(3) Each variant entry is normalized according to the normalization algorithm proposed by Tan et al. [2].

The same normalization steps are also applied to the included annotation databases to ensure, that the assignment of annotation information to the individual variants is reliable and consistent.

2 Data security measures

As a software like AMLVaran works with sensible medical data, data security is a priority that has to be kept in mind.

First of all, unlike commercial web-based tools (e.g. VarSome [3]), AMLVaran is open-source software, and is intended to be installed locally at the sequencing facility. This ensures that users do fully retain control over their data.

The web application itself has been implemented with particular consideration to recommended best practices of security that should avoid typical attacking vectors. This includes encrypted transfer of data between client and server, server-sided filtering of user input, prevention of SQL injections by use of prepared statements, user management with session authentication, salted hashes for password encryption, automatic logout after inactivity etc. (cf. [4]).

Nevertheless, also the web-server has to be secured against intrusions. Common measures of IT security need to be taken by the system's administrator, when the software is installed. This involves, but is not limited to, packet-filters between the server and the internet, enforcing SSL-encrypted data transfer, preventing unauthorized (physical) access to the server, ensuring regular updates of critical components, providing regular backups, etc. (cf. [5]).

Apart from this, legal or ethical questions may arise, regarding the handling of patients and their data, as discussed previously [6, 7]. Patient consent must be obtained according to ethical standards and legal requirements [8].

3 Lessons learned from usability studies

During the first user study with AMLVaran's prototype, the following six aspects were identified to require improvement:

(a) Genomic coordinates and nucleotide changes are only relevant in certain contexts. While commonly used in bioinformatics, expert feedback revealed, that these annotations have little meaning for the average medical user. Clinical users requested a presentation on protein and amino acid level.

(b) Abbreviations that are common in the context of variant calling tools, e.g. AF for Allele Frequency, were not familiar to all users. Abbreviations must therefore be avoided or explained to achieve sufficient usability of the program.

(c) Annotation sources, which provide orientation for an unknown variant's pathogenicity, can result in a multitude of numbers and tend to confuse users. Annotation information should therefore be presented in a structured way, including short information texts that help to correctly interpret the presented scores.

(d) Prioritization of variant lists requires to take a lot of information into account. This tends to be a complex task for a human being, an issue that is further aggravated for users that are primarily trained on the medical or biological field. The software must therefore support the users in the complex process of decision finding.

(e) The importance of "no information" is not inherently clear, and relevance of a lack of information has to be emphasized. In sequencing studies, arbitrary regions can lack sufficient coverage, or show contradicting information. In such cases, it is possible, that no reliable conclusion about the presence or absence of a certain variation can be made. It must therefore be made transparent for the users whether a "negative" result is dependable or not.

(f) Large result pages, with require a lot of scrolling, produced stress for the users. A simple-to-complex interface, with a rough overview and the option to open sub-windows with more detailed information on a single mouse-click was better accepted by the participants of the final study.

4 Considerations on a non-binary way of classification

The appreci8 filtering scheme outputs not only a proposed classification of each variant into one of the categories "artifact", "polymorphism" and "probably true", but also two numeric scores, which are calculated from a lot of criteria, including (but not limited to) the number of variant calling tools that had detected the variant. As depicted in figure 1, a higher Artifact score correlates with an increased certainty of a variant being an artifact (a), and a higher Polymorphism score increases the probability that a variant is not pathogenic (b). It could be examined, whether a probability for the reliability of the classification can be derived from these scores.

From the analyzed test datasets, for which validation data is available, a relative frequency of confirmed artifacts resp. polymorphisms can be calculated for each score value, as shown in table 1.

From a statistical point of view, with a large amount of validation data, a probability measure could be estimated from those relative frequencies, giving a measure of certainty whether the proposed classification of the variant may be true or false.

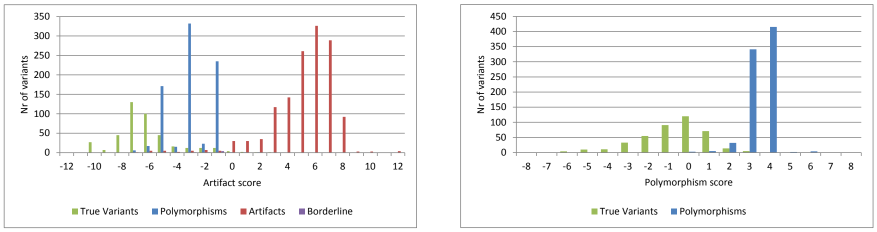

(a) (b)

Figure 1 Correlation between variant types and scores. (a) Artifact scores. For each possible value of Artifact score, the number of variants are displayed that have been validated as true resp. as artifact. True variants (and polymorphisms) tend to have lower artifact scores, while most confirmed artifacts are linked with higher values of Artifact score. Borderline cases are observed primarily with artifact score -1. (b) Polymorphism scores. All confirmed variants (artifacts have been excluded), are grouped by their assigned Polymorphism score. Confirmed polymorphisms show higher Polymorphism score than true variants.

Table 1 Probabilities of variant classification, estimated from dataset MDS-1.

(a)

Artifact score	-12	-11	-10	-9	-8	-7	-6	-5	-4	-3	-2	-1	0
Nr of variants	0	0	27	7	45	137	122	221	33	350	43	256	35
Conf. artifacts	0	0	0	0	0	1	5	5	2	5	7	5	30
Borderline var.	0	0	0	0	0	0	0	0	0	1	1	4	0
Rel. frequency	0	0	0	0	0	0.01	0.04	0.02	0.06	0.01	0.16	0.02	0.86

Artifact score	1	2	3	4	5	6	7	8	9	10	11	12
Nr of variants	33	37	118	142	261	326	289	92	3	3	0	4
Conf. artifacts	30	35	117	142	261	326	289	92	3	3	0	4
Borderline var.	0	0	0	0	0	0	0	0	0	0	0	0
Rel. frequency	0.91	0.95	0.99	1.00	1.00	1.00	1.00	1.00	1.00	1.00	1.00	1.00

(b)

Polymorphism score	-8	-7	-6	-5	-4	-3	-2	-1
Nr of confirmed variants	0	0	4	10	11	33	55	91
Confirmed polymorphisms	0	0	0	0	0	0	0	0
Relative frequency	0.00	0.00	0.00	0.00	0.00	0.00	0.00	0.00

Polymorphism score	0	1	2	3	4	5	6	7	8
Nr of confirmed variants	123	76	46	346	415	2	4	0	0
Confirmed polymorphisms	3	5	32	341	415	2	4	0	0
Relative frequency	0.02	0.07	0.70	0.99	1.00	1.00	1.00	1.00	1.00

From a medical point of view, however, there are two drawbacks that have to be taken into account:

(1) Since the mutational pattern of a patient may be distinctive for the type of treatment, there is a need for a clear decision, whether a variant is present or not. The process of decision, including applied thresholds, should be standardized and reproducible. The appreci8 pipeline uses a threshold of 3 (for artifact score) and 0 (for polymorphism score). Both of them have been optimized and validated with several datasets, as presented in [9]. Nevertheless, AMLVaran's analysis algorithm is flexible enough to allow custom adaptations, depending on the dataset and lab protocols.

(2) We agree with the perception of the ACMG workgroup, who stated, that they do not see any medical indication for assigning fixed weightings to single variant assessment criteria [10]. Especially, they could not agree on such values. Therefore, we feel, that giving out probabilistic scores could rather lead to the confusion of

Table 2 Runtime for complete analysis of an average sample (546 MB bam-file, using 4 CPU cores).

	CPU (using 4 cores)	RAM	runtime	in hours
Dedicated virtual server	Intel Xeon E5620 @ 2.40GHz	32 GB	6,241 sec	1:44 h
Shared server	Intel Xeon E5-2695 v4 @ 2.10GHz	500 GB	3,299 sec	55 min
Consumer workstation	Intel Core i5-4590 @3.30GHz	8 GB	2,750 sec	46 min

users who might take those as a valid probability for a certain variant to be present in a single patient, which would not be legitimated from data.

Nevertheless, Artifact and Polymorphism scores are displayed, giving the user an orientation, how reliable the classification of each variant can be considered.

5 Runtime evaluation

AMLVaran was installed on a virtual server with an Intel Xeon E5620 @2.4 GHz CPU with 32 GB RAM, under the Operating System Debian 8.7 with Linux kernel v3.16.0-4 (amd64), and on a desktop workstation with an Intel Core i5-4590 Quad CPU @3.3 GHz, 8 GB RAM, Ubuntu Desktop 18.04.2 LTS, and 500 GB of disk storage. The back-end consisted of an Apache web server and MySQL database (version 5.5.57).

The resulting runtime was sufficient for practical use in both cases. A comparison of the runtime needed to perform the analysis for an average sample (546 MB bam-file) is shown in Table 2.

6 Processing WES sequencing samples

Although AMLVaran has been developed and optimized for targeted NGS sequencing, sometimes there is a demand for processing whole-exome (WES) data as well.

Technically, the generic variant calling pipeline is flexible enough to process even WES data. However, the resulting variant lists will get by orders of magnitudes longer than with targeted data, which imposes some challenges for downstream processing steps. Furthermore, the invocation of eight different variant callers may be impracticable, since this would lead to an inacceptable total runtime.

AMLVaran was tested with some whole-exome samples from cancer and germline data. Since a combination of eight callers seems not feasibly with WES data, the variant calling pipeline was reduced to a less complex configuration using (a) only one caller (GATK which is one of the most common tools), (b) two callers (GATK and VarScan) and (c) three callers (GATK, VarScan and FreeBayes, which is the combination proposed by CoVaCS [11]). Table 3 shows the results (number of called variants and runtimes) with a WES sample containing 80 million reads, with an average coverage of $84x$ in a bam-file of 5.9 GB size. All validated mutations have been detected by AMLVaran with these samples.

The generic variant calling pipeline successfully processed the WES data. However, dealing and filtering lists with thousands or ten-thousands of variants is not really feasible and user-friendly within the browser-based user-interface. Additionally, depending on the selected web-browser, considerable performance issues have been experienced on client side with loading and filtering variant lists of WES samples. This is, why we would recommend AMLVaran only to a limited extent for the analysis of complete WES samples.

Since AMLVaran has been developed with a clear focus on diagnostic recommendations, and only a small part of the existing annotation data has medical relevance

Table 3 Results for WES sequencing sample.

(a) Nr of called variants

	raw calls	region filter	SNPeff filter	basic filter
GATK	45,105	45,014	11,664	7,973
GATK, VarScan	65,404	45,944	11,891	8,102
GATK, VarScan, FreeBayes	632,130	526,946	261,308	8,436

(b) Runtime measure

	combining results	region filter	SNPeff filter	read quality	total runtime (excl. callers)
GATK	3:36 min	2 sec	1:35 min	1:19 min	6:39 min
GATK, VarScan	14:26 min	3 sec	1:36 min	2:04 min	18:17 min
GATK, VarScan, FreeBayes	39:31 min*	29 sec	2:17 min	31:40 min	1:20 hours

* Combination of genotypes was disabled to process half a million of FreeBayes variants at reasonable performance.

or an assured relevance to therapy, clinical users typically will be interested only in selected genes. AMLVaran provides an optimized mode of processing for such an use case: When uploading a WES sample, the analysis process can be restricted to relevant genes or regions, either by selecting one of the predefined target panels, or by uploading a custom bed-file. We recommend this form of processing WES data, since it reduces the number of variants of unknown significance in the output list, and thus noticeably increases processing speed and website performance.

Author details

References

1. Danecek, P., Auton, A., Abecasis, G., Albers, C.A., Banks, E., DePristo, M.A., Handsaker, R.E., Lunter, G., Marth, G.T., Sherry, S.T., McVean, G., Durbin, R.: The variant call format and VCFtools. Bioinformatics (Oxford, England) **27**(15), 2156–2158 (2011). doi:10.1093/bioinformatics/btr330
2. Tan, A., Abecasis, G.R., Kang, H.M.: Unified representation of genetic variants. Bioinformatics (Oxford, England) **31**(13), 2202–2204 (2015). doi:10.1093/bioinformatics/btv112
3. Kopanos, C., Tsiolkas, V., Kouris, A., Chapple, C.E., Albarca Aguilera, M., Meyer, R., Massouras, A.: VarSome: The human genomic variant search engine. Bioinformatics (Oxford, England) **35**(11), 1978–1980 (2019). doi:10.1093/bioinformatics/bty897
4. Federal Office for Information Security: Security of web applications - Catalogue of measures and best practices [Sicherheit von Webanwendungen - Maßnahmenkatalog und Best Practices] (2006). `https://www.bsi.bund.de/DE/Publikationen/Studien/Websec/index_htm.html` Accessed Nov. 27, 2019
5. Federal Office for Information Security: Server protection (ISi-Server) [Absicherung eines Servers] (2013). `https://www.bsi.bund.de/DE/Themen/StandardsKriterien/ISi-Reihe/ISi-Server/server_node.html` Accessed Nov. 27, 2019
6. Joly, Y., Saulnier, K.M., Osien, G., Knoppers, B.M.: The ethical framing of personalized medicine. Current opinion in allergy and clinical immunology **14**(5), 404–408 (2014). doi:10.1097/ACI.0000000000000091
7. Salari, P., Larijani, B.: Ethical Issues Surrounding Personalized Medicine: A Literature Review. Acta medica Iranica **55**(3), 209–217 (2017)
8. Duardo-Sánchez, A., de Miguel Beriain, I.: Personalized Medicine and Medicinal Chemistry: Toward a Legal Framework in the European Union. Current topics in medicinal chemistry **18**(25), 2165–2173 (2018). doi:10.2174/1568026619666181120144213
9. Sandmann, S., Karimi, M., de Graaf, A.O., Rohde, C., Göllner, S., Varghese, J., Ernsting, J., Walldin, G., van der Reijden, B.A., Müller-Tidow, C., Malcovati, L., Hellström-Lindberg, E., Jansen, J.H., Dugas, M.: appreci8: A Pipeline for Precise Variant Calling Integrating 8 Tools. Bioinformatics (Oxford, England) (2018). doi:10.1093/bioinformatics/bty518
10. Richards, S., Aziz, N., Bale, S., Bick, D., Das, S., Gastier-Foster, J., Grody, W.W., Hegde, M., Lyon, E., Spector, E., Voelkerding, K., Rehm, H.L.: Standards and guidelines for the interpretation of sequence variants: A joint consensus recommendation of the American College of Medical Genetics and Genomics and the Association for Molecular Pathology. Genetics in medicine : official journal of the American College of Medical Genetics **17**(5), 405–424 (2015). doi:10.1038/gim.2015.30
11. Chiara, M., Gioiosa, S., Chillemi, G., D'Antonio, M., Flati, T., Picardi, E., Zambelli, F., Horner, D.S., Pesole, G., Castrignanò, T.: CoVaCS: A consensus variant calling system. BMC genomics **19**(1), 120 (2018). doi:10.1186/s12864-018-4508-1
