# Supplementary material for: AMLVaran: a software approach to implement variant analysis of targeted NGS sequencing data in an oncological care setting
Source: BMC Med Genomics. 2020 Feb 4;13:17. doi: 10.1186/s12920-020-0668-3 (PMC7001226; doi:10.1186/s12920-020-0668-3)
Supplement: Supplementary file 2 — Additional file 2 A PDF document, giving basic installation and usage instructions for the software. [file 12920_2020_668_MOESM2_ESM.pdf]

AMLVaran Quick Start Guide

1. Demo logins

We provide three data sets with a total of 404 samples, which we analyzed in the paper, for demonstration purposes. The data sets include different target panels and are evaluated with different hotspot information.

Dataset AML-1 (AML, 119 samples)	User: training	Password: Halle2015
Dataset MDS-1 (MDS, 237 samples)	User: test1	Password: Sweden2017
Dataset MDS-2 (MDS, 46 samples)	User: test2	Password: Sweden2017

Please click on “LogIn” in the top right corner and enter your personal or some of the demo credentials.

2. Selecting a sample

After logging in, you will see an overview of all patients and samples assigned to your user account.

On the right side all analyzed samples are listed. These can be sorted by clicking on the column headings (e.g. the most recent samples at the top).

On the left side the registered patients are listed. By clicking on a patient, the sample list can be filtered to display only this patient’s samples.

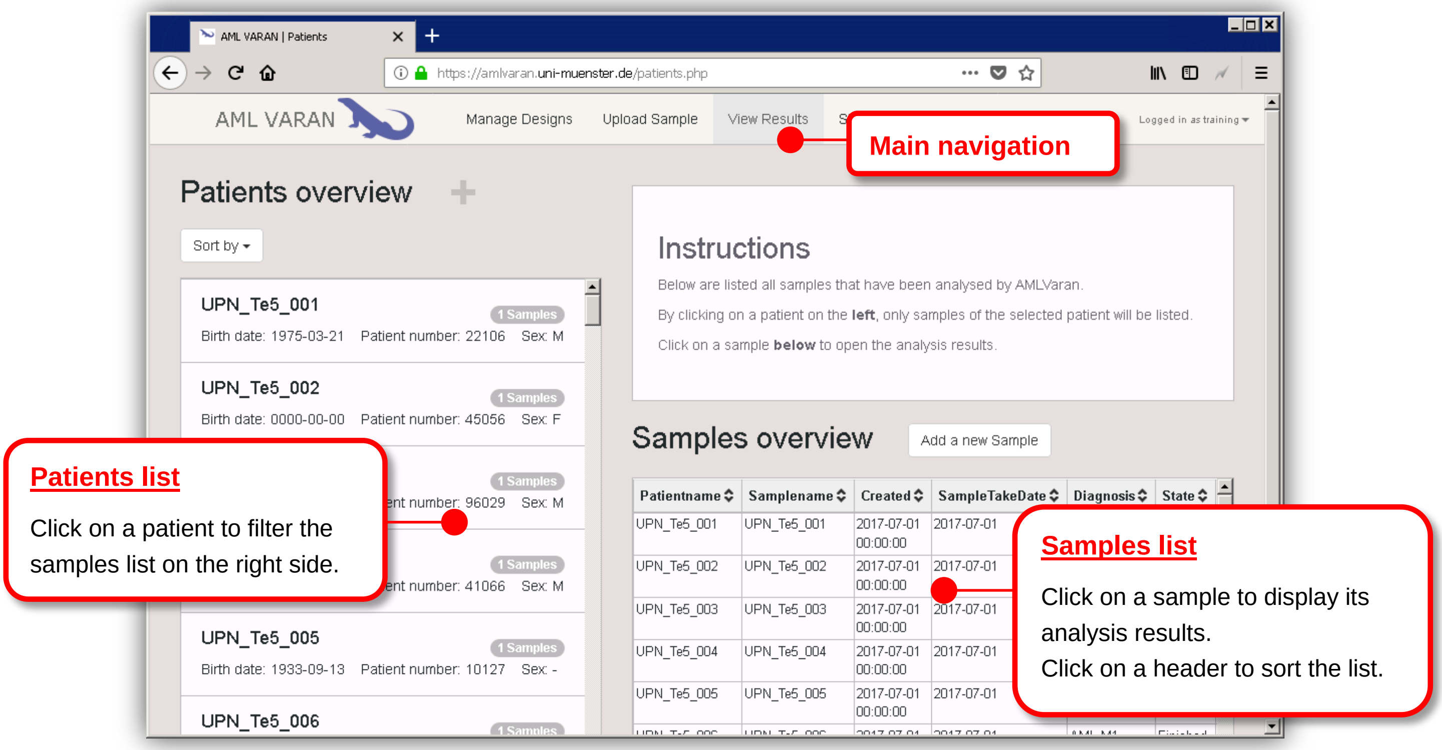

3. Inspecting results of a sample

After selecting a sample, the interactive results page for this sample will be shown.

The results page is divided into 4 functional areas (Sample Info, Hotspots, Variant Inspector, Genome Browser), which are briefly presented below:

Sample Info

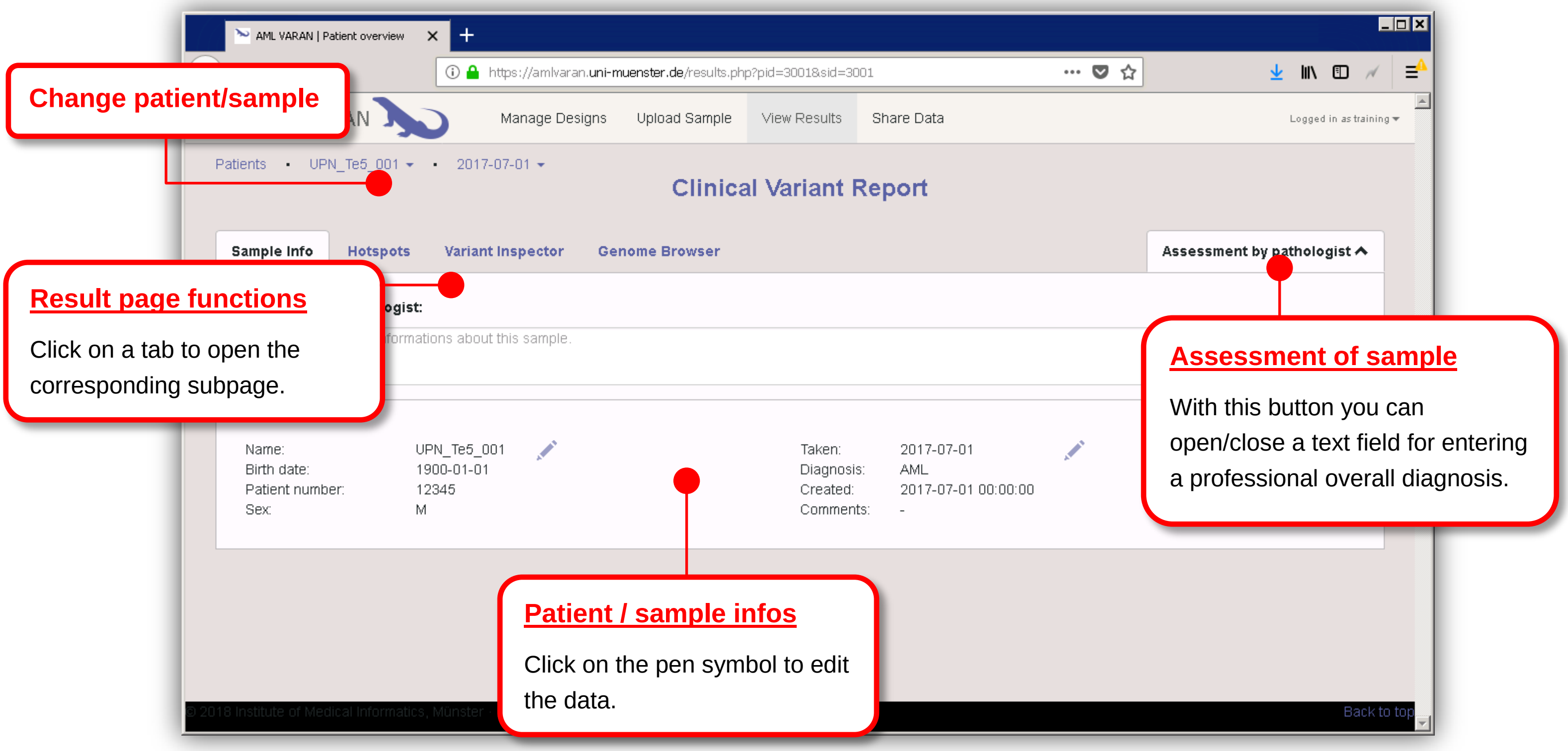

Hotspots

An overview of the known, predefined driver mutations is displayed.

Each rectangle symbolizes a genomic range. If a mutation has been discovered in this range that has not been classified as artifact or polymorphism, the rectangle is colored red. If no (real) mutation was found in the specified area, the rectangle is green. If no mutation has been detected, but the coverage is not sufficient for a certain exclusion, the rectangle turns yellow.

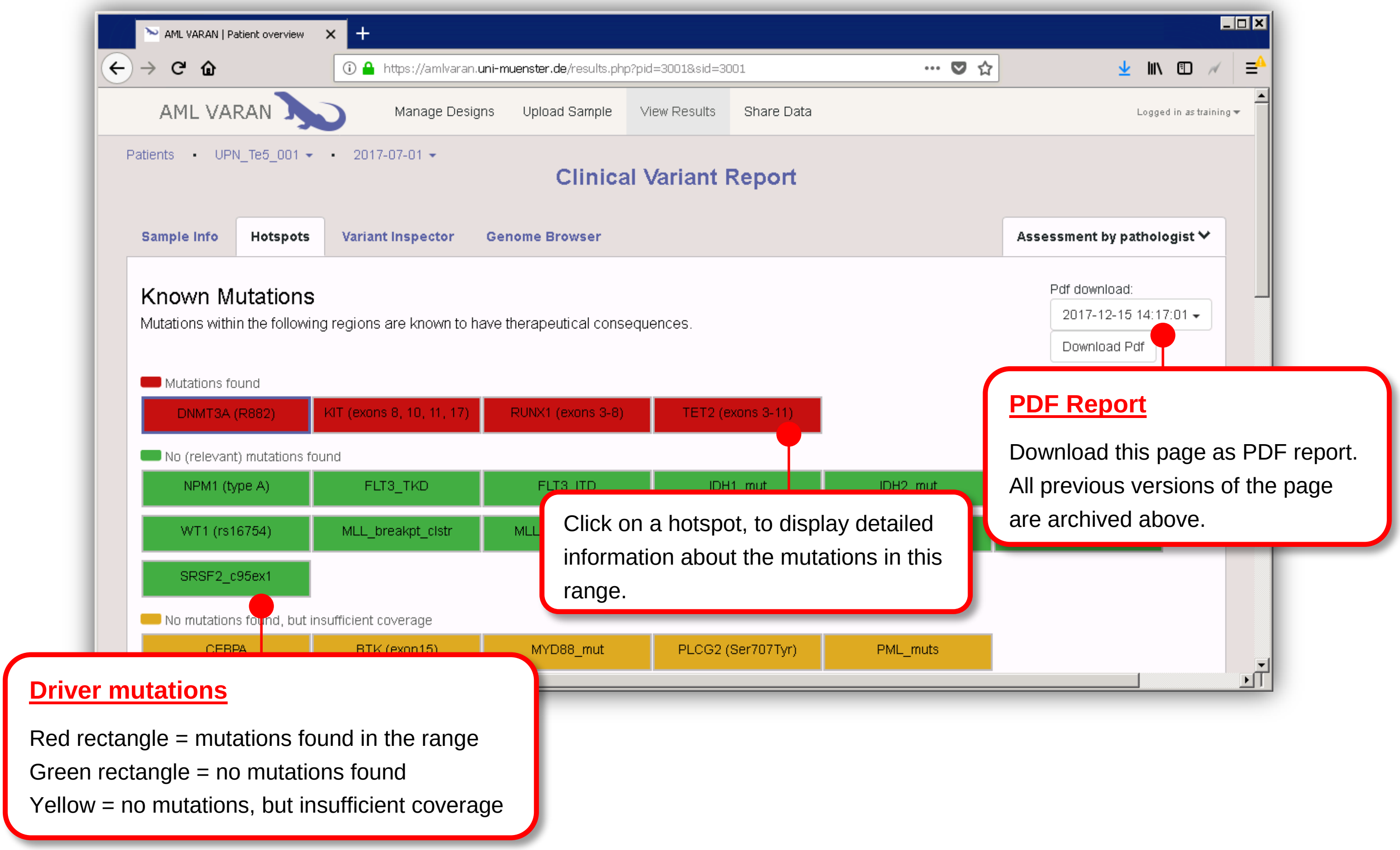

When clicking on a hotspot region, a detailed view is presented:

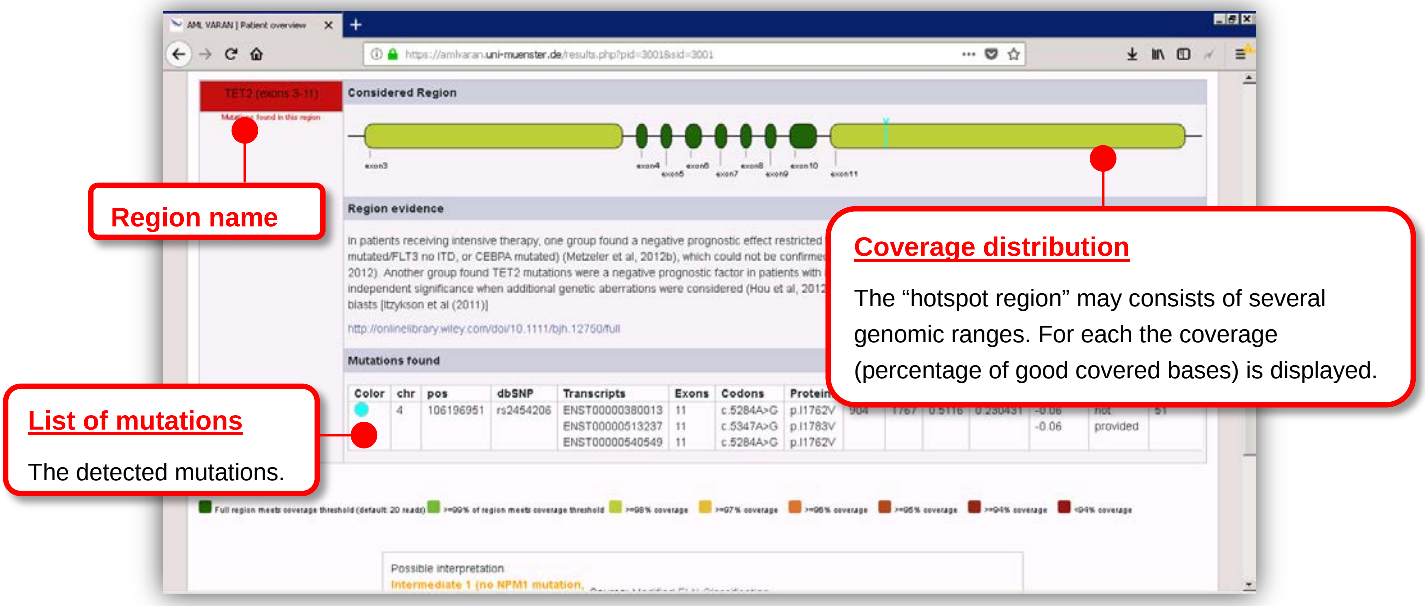

Variant Inspector

Displays all variants detected in the current sample, and offers numerous dynamic filtering and sorting settings. Further details are explained in the following chapter.

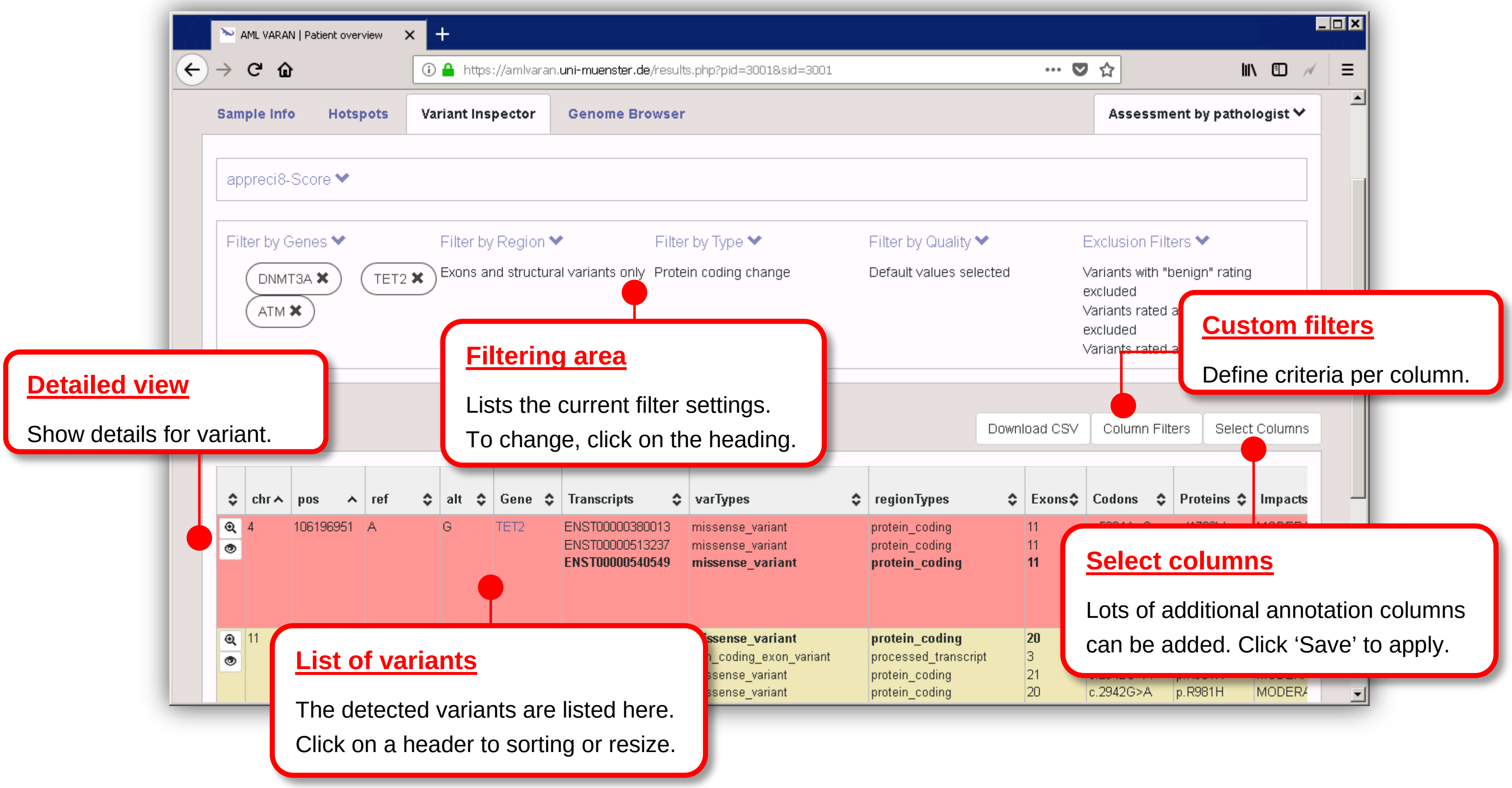

Genome Browser

Useful for inspect the sequencing raw data. The individual reads are displayed and any deviations from the reference genome are highlighted in color. Move the displayed viewport with the mouse or use the mouse wheel to zoom in or out.

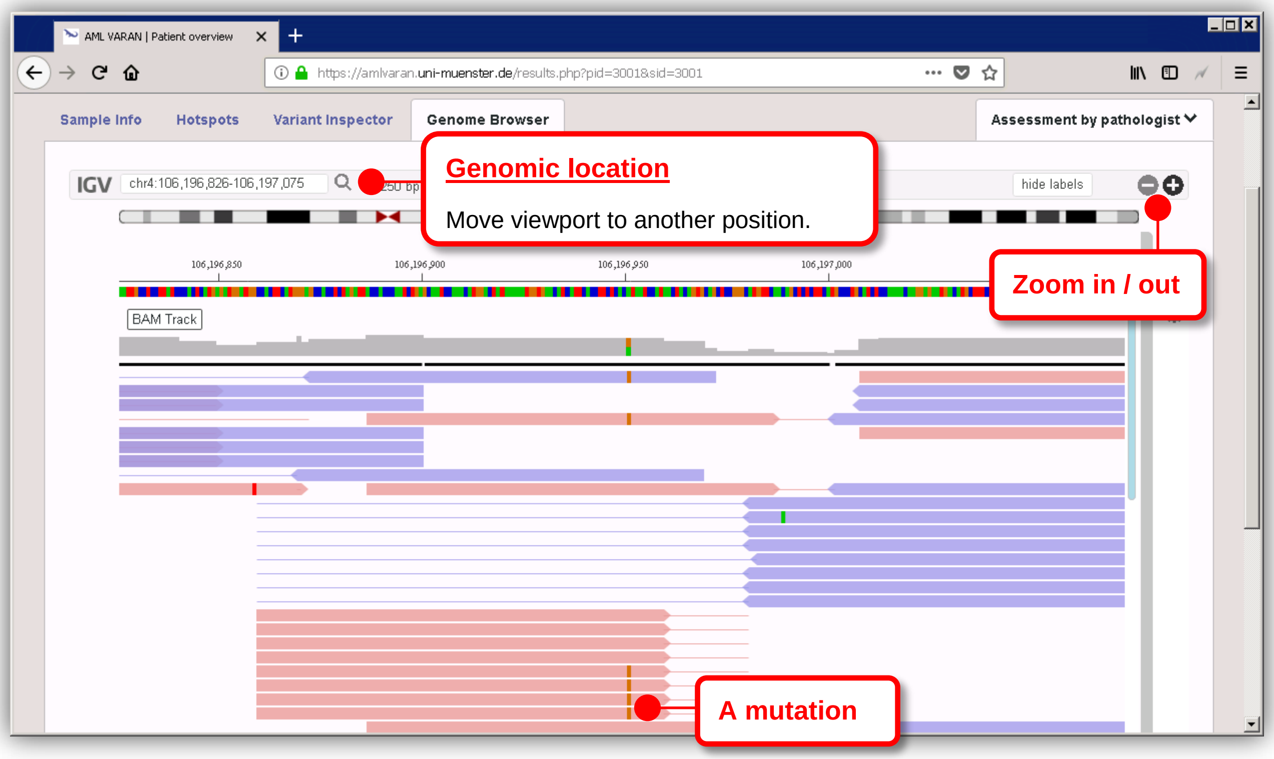

4. Using the Mutation Explorer

Filtering, sorting

The variant list can be adapted to your needs by

(a) Selecting the columns you might be interested in (lots of additional annotation columns are ready to be activated)
(b) Sorting the variants by one or more selected columns. Click on the column header to sort by this column. Click twice to change the sorting order. Hold CTRL when clicking on column to sort by more than one entry.
(c) Changing the column sizes: Just click and hold the header boundaries to change column size.

The variant list can also be filtered by predefined criteria, which will be explained on the picture:

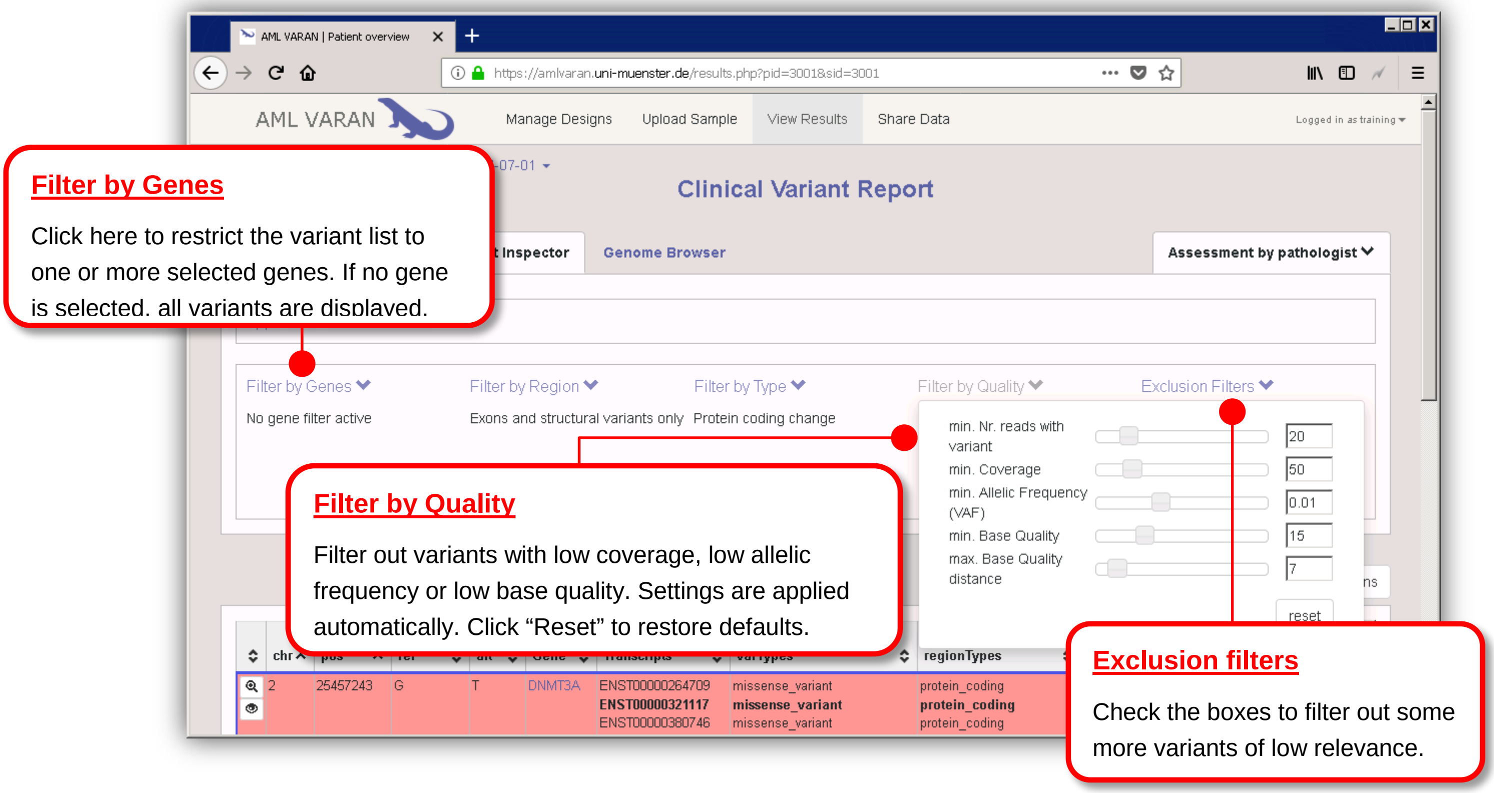

Custom Filters

Click on Custom filters to define your own criteria for each column. You can use a search text (e.g. "missense") which must be part of the column text, or a numeric operation (e.g. ">0.5"). Several criteria can be combined using logical operators (AND; OR; NOT). If you want to include / exclude columns without content, please use double quotes with nothing between ("").

Detailed view

Click on the magnifier icon in the leftmost column of a variant to open the detailed view for this variation. The detailed view is divided into several tabs, which focus on different annotations classifying a variant.

(a) **Gene information:** Displays the full name of the gene, some short summaries about its function and pathways and provides some web-links with further information about the gene.

(b) **Detected variant:** Shows the genomic and protein-based location of the mutation, the type of the aberration and some key figures about the sequencing quality within the current sample (e.g. Allelic Frequency, Coverage, Base Quality, Strand Bias…)

(c) **Databases information:** Shows annotations for this variant as provided by widely used clinical research databases. The ClinVar entries provide an orientation whether the variant has been proven to be clinically relevant for certain diseases. COSMIC is a collection of several sequencing projects and gives an overview on the diseases in connection with which this mutation is frequently reported. 1000 Genomes, ESP6500, ExAC etc. are sequencing projects that examined healthy humans. The table shows the frequencies with which this mutation occurred in healthy humans. If this value is high (e.g. ≥0.01) in one or more projects this may be a sign of benignity.

(d) **Functional prediction:** This page shows the results of various functional prediction tools trying to estimate the mutation's degree of influence on protein synthesis. We also provide a graphical ranked score representation, which depicts the rank of the current variant's pathogenicity in comparison to all other possible non-synonymous protein-coding variants assessed with the same functional prediction tool.

(e) **Literature references:** In this tab all literature references for the current gene from the CiVIC database are listed. You can find literature references for a specific mutation (e.g. "R882" in the left column) or about general mutations within the gene ("MUTATION" in the left column). Click on "References" to show the single publications.

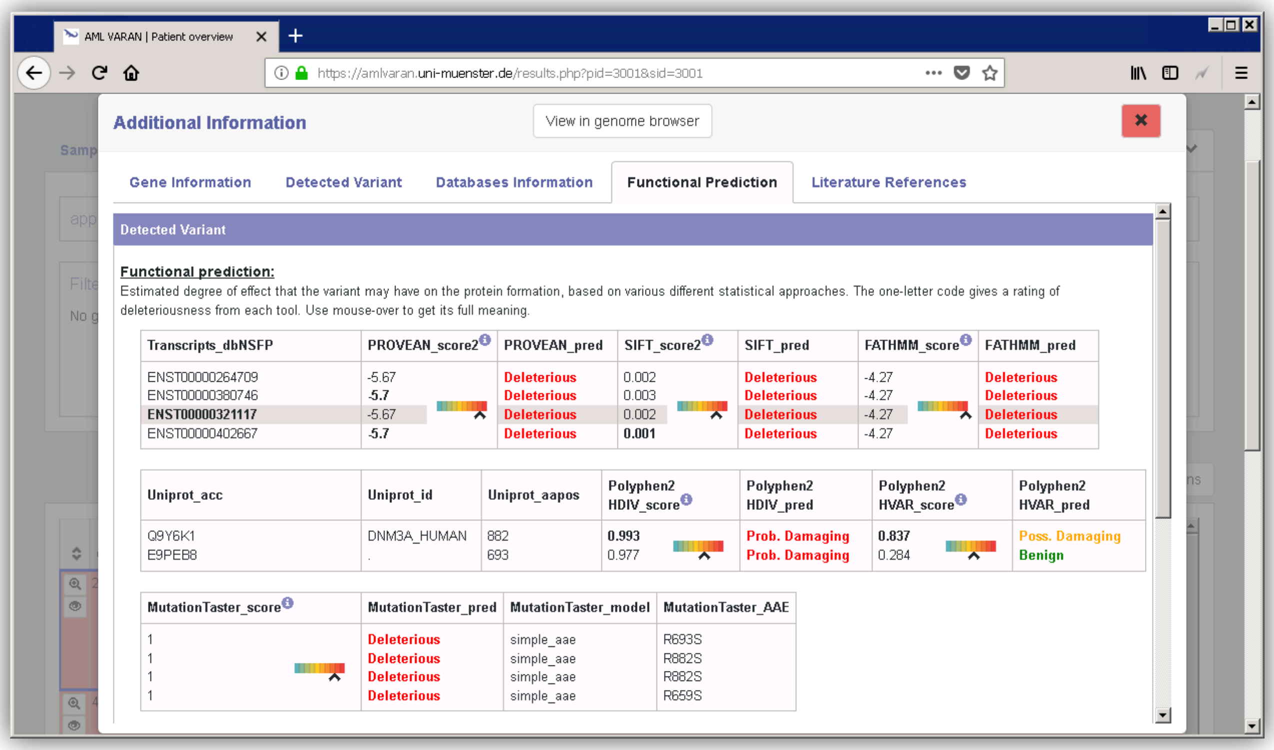

5. (Re-)Calculate variant scores

By default, an artifact- and a polymorphism-score is calculated from all the variants that have been called by any of the combined variant callers, by means of the appreci8 algorithm.

The score calculation scheme can be inspected and dynamically customized by clicking on the button “appreci8-Score”. You will then get a graphical interface, showing each criterion that may contribute to one of the two scores. Each of the criteria can be enabled or disabled, the thresholds can be adapted or the contribution value can be increased or decreased.

With clicking on “Calculate scores” the artifact and polymorphism scores will be recalculated for all variants in the current sample. Depending on the filter settings, this can lead to more (or less) "real" mutations being displayed in the variant list than before.

You can also return to the default settings at any time by pressing "Reset".

Note: The customized filter scheme only affects the Variant Inspector page. The clinical report and the Hotspot overview will not be changed.

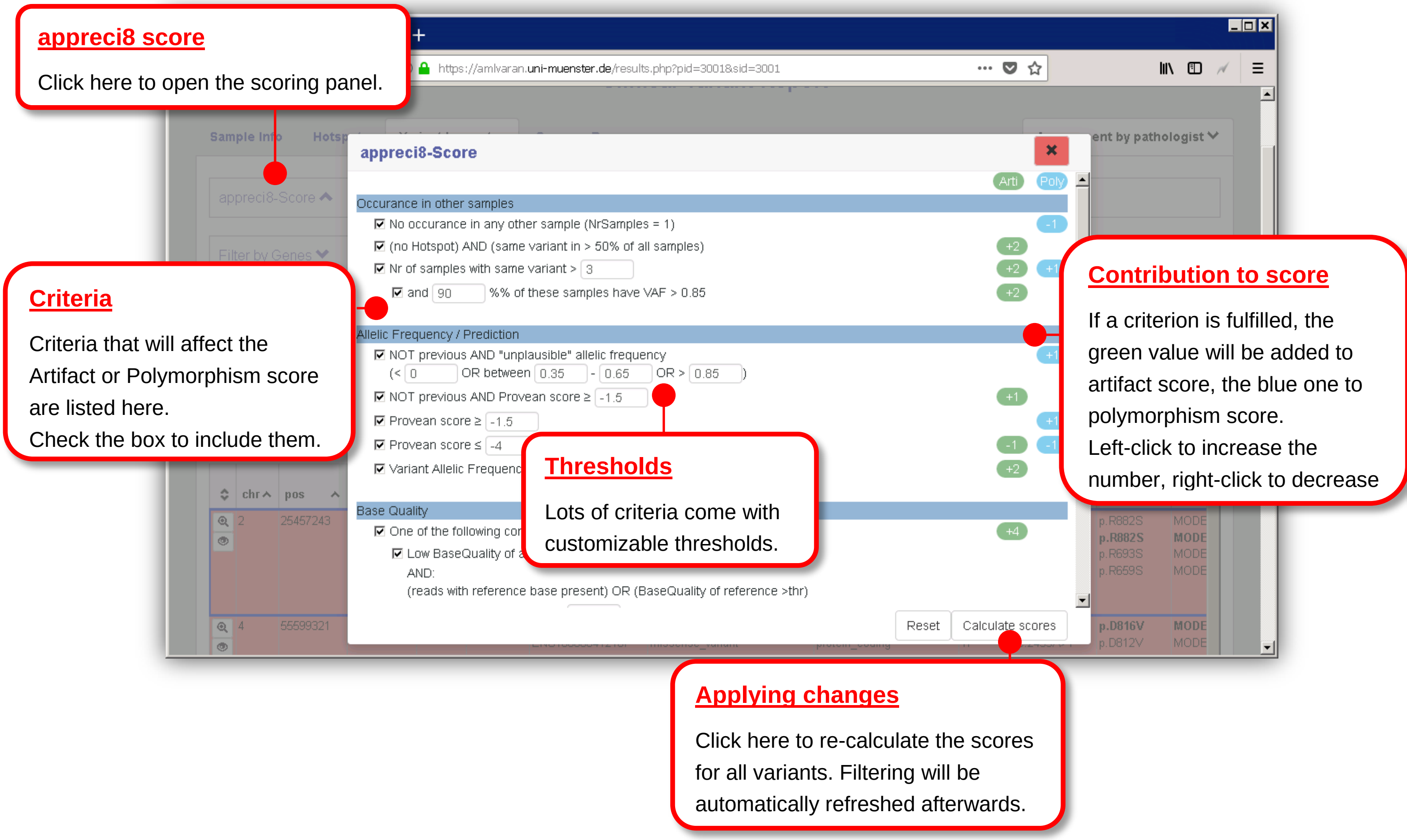

Using a custom score calculation scheme

It is also possible to include your own, fully customized score calculation scheme into AMLVaran. We specified a simple and powerful scoring scheme definition language in JSON format. By this means custom score calculation schemes can easily be created by everyone. They are even universally usable not only in websites but also in Excel, R and standalone. Just use our interpreters, available from http://annoserv.uni-muenster.de.

6. Uploading a sample

(Please note, that the demo users, as well as newly registered users are not allowed to upload samples! This is necessary in order to protect our servers from abuse. If you want to try out the analysis pipeline, please drop an e-mail to c.wuensch@uni-muenster.de telling me your account name and asking for unlocking the upload functions.)

There are two ways to upload a sample for analysis: (a) click on “Upload sample” in the main navigation bar or (b) click on “Add a new sample” in the samples overview.

You will then be guided through the process by a simple HTML form asking you to enter the necessary information.

First you have to select a patient, to whom the sample belongs (several samples can be assigned to one patient, e.g. for follow-up studies). Just enter the name or pseudonym of the patient. If there already exists a corresponding entry, it will be suggested automatically. If not, a new patient will be created. Optionally you can specify some more information about the patient, such as date of birth, but that is not required.

Also you will be asked to select a target panel design that shall be used for the analysis. There are just our AML resp. MDS consensus panels preconfigured. If your data has been sequenced with another target kit, you will have to create a new design before (see the following section for instructions).

Finally you have to upload the raw data of the sample to be analyzed. AMLVaran can accept unaligned filed in fastq format, aligned files in bam-format as well as variant lists in vcf format. The type of data is automatically detected from the filename extension.

It is strongly recommended, however, to use only pre-aligned bam-files for upload! The reason is that raw fastq data is little standardized, often needs some preprocessing like trimming, and in case of paired end data, it is difficult to create a single fastq-file that can be uploaded. Our published pipeline does include some preprocessing steps for fastq-files and has been successfully tested with certain files, but it most likely would need some adaptation for fastq-files from different sources.
Also vcf files are supported but not recommended as input, because then all the quality info from the bam file will be missing.

After uploading the sample, the analysis pipeline will be automatically started, as soon as there is a free slot on our worker server.

Creating a custom target panel

In case an analysis of a targeted NGS sample with a target panel that is not already listed in AMLVaran’s list of designs shall be performed, you have to create a new design fist.

To do so, please click on “Manage Designs” in the main navigation bar. Then you will be prompted to specify a name for your custom design and to upload the corresponding list of target regions in bed format. After doing so, your new design will be listed in the designs list, and can be used for the analysis of new samples.

Processing WES samples

In a clinical setting, users typically are interested in selected genes or regions, which are known to be relevant for therapy. If not all genes are of interest, it is recommended, not to select the “Agilent SureSelect V5 WES Panel” for analysis, but a (predefined or custom) target panel. This will significantly improve performance of analysis pipeline and interactive filtering.

7. Initial Setup / Installation

The easiest method of installing AMLVaran is to use our preconfigured Docker script, which creates three Docker containers (Webserver, Database and Worker), starts them and provides the network connection between them, so the system can be run "out-of-the-box".

1. Please use a recent Linux environment, e.g. Debian or Ubuntu and make sure, that you have got at least 4 CPU cores, 8 GB of RAM and 500 GB free disk space.
2. Install Git, Docker, docker-compose and dependencies via your system's packet manager:
   ```
   sudo apt-get install git
   sudo apt-get install docker.io
   sudo apt-get install docker-compose
   ```
3. Checkout the source-code from Git repository:
   ```
   git clone https://github.com/cwuensch/AMLVaran.git amlvaran
   ```
4. If you want to use GATK 3 as a variant caller, please download it and place GenomeAnalysisTK.jar into the amlvaran root directory:
   ```
   wget https://software.broadinstitute.org/gatk/download/auth?package=GATK-archive&version=3.3-0-g37228af
   tar -xjvf GenomeAnalysisTK-3.3-0-g37228af.tar.bz2 amlvaran
   ```
5. The script `Docker_start.sh` guides you through the process of assembling and configuration of the software components.

During the installation, required tools and a pre-populated database will be downloaded and installed within the Docker containers. Hence, the installation may take a few hours.

After, the process is complete, the AMLVaran system will be up and running. You can access the web-interface from your browser at http://localhost:80.

CAVE: The Docker installation does NOT provide a secure webserver. If you want to use the system productively, we strongly recommend to filter port 80 in your firewall and to provide a public webserver, which uses a SSL-certificate and encrypted data transfer, and forward the AMLVaran output through your secure webserver.

8. Manual installation

If you cannot use the preconfigured installation method via Docker (which is recommended), you will find a description of the individual steps below, that are required to install the entire AMLVaran platform on your own web server (or on separate servers for the web interface, the database and the variant analysis pipeline).

Setting up webserver

A Linux PC with Apache web server including PHP and MySQL support is required to run the web interface. Install the web server according to the manufacturer's instructions.

In the following, we assume that the web server will be set up such that the public files are located in the directory `/var/www`.

The uploaded samples will then be stored under `/var/samples` by default. Please make sure that there is enough space available in this folder and that PHP has read and write access to this folder. The worker PC running the variant analysis pipeline must also have read and write access to the samples folder.

(CAVE: The samples folder must not be located within the public area of the web server!)

Setting up database

For the operation of AMLVaran a MySQL database is required, which holds the usual information (registered users, patients, samples, etc.), the analysis results as well as the pre-processed annotation databases. For the annotation databases, storage space of approx. 150 GB should be planned, in addition, the database should provide further storage for the analysis results.

Please install a MySQL server according to the manufacturer's specifications and create an empty database. (Please make sure to define an individual user name and password!)

The database server must be accessible both from the web server and from the worker PC(s) on which the variant analysis pipeline is executed, and must allow read and write access.

The table structure required for AMLVaran must be created in the empty database. This is achieved by importing the provided SQL dump, which creates the empty DB structure. In addition, we recommend downloading and importing our current build of preprocessed annotation databases (also provided as SQL dump).

Obtaining the source code

The source code for AMLVaran is provided via GitHub at https://github.com/cwuensch/AMLVaran.

It consists of two parts:

The folder `/www` contains all data required for the generation of the web interface. These must be checked out into the public area of the web server (under `/var/www`). The `/samples` folder is used for recording the samples and must provide sufficient storage space.

The pipeline folder contains the scripts that are needed to analyze the samples. The pipeline is independent of the web interface and can be run on the web server itself or on one or more separate (identical) servers. Each of these servers requires read and write access to the samples folder and the database.

The pipeline is programmed as bash scripts and in Python, and calls other third-party tools. The installation of Python is required. Further configuration is described below.

Prerequisites

The following third-party tools must be installed on the server that executes the variant analysis pipeline:

- Python 2.7.1 or Python 3.2 or higher
- Java JDK 1.8 or higher
- VariantTools 2.7: http://varianttools.sourceforge.net/
- samtools 1.3: http://www.htslib.org/
- vcftools 0.1.13: https://vcftools.github.io/index.html
- SNPeff: http://snpeff.sourceforge.net/
- bam-readcount: https://github.com/genome/bam-readcount
- *Provean: http://provean.jcvi.org/index.php [optional]*
- *bwa 0.7.12: http://bio-bwa.sourceforge.net/ [only for alignment]*
- *trim_galore 0.4.1: https://www.bioinformatics.babraham.ac.uk/projects/trim_galore/ [only for preprocessing]*

- *CutAdapt 1.9.1: https://cutadapt.readthedocs.io/en/stable/ [only for preprocessing]*

In addition, the following resources must be provided on the worker server:

- reference genome (e.g. Homo_sapiens.GRCh37.67.dna.chromosome.all.fasta) + .fai index
- bwa-index for reference genome (must be created via "bwa index xy.fasta")
- peptide files for Provean (the AA sequence for each protein, optional)

Since the above files may require a lot of storage space, it is recommended to store them centrally and to mount them in each worker server (if more than one). This keeps the footprint of the worker servers small.

Configuration (Paths, Logins)

Webserver

On the web server must be provided:

a) access to the samples folder, which is located under `/var/samples` by default. The data can be stored locally on the server, or via NFS mount on a file server. However, read and write access from PHP must be guaranteed.
CAVE: The samples folder must not be located within the public www folder of the webserver!

b) login credentials for the MySQL database must be entered in the file `/www/inc/constants.inc.php`. There must be read and write access to the database.

Worker server

On the server(s) that execute the variant analysis pipeline (several worker servers of the same type can be used – If there is more than one, the individual workers are automatically coordinated with each other), the following prerequisites must be fulfilled:

a) Installation or provision of the tools and resources described under Prerequisites.

b) Installation of the variant caller tools to be used and configuration as described under Adding a variant caller.

c) Reading and writing access to the samples folder and the database.

d) The access data to the MySQL database must be entered in `~/.my.cnf` for the user running the pipeline.

e) The paths to the folders and tools used are entered in the /pipeline/Config.sh file.

f) A daemon must be set up, which starts `PipelineDB.sh` once when booting the PC. This checks then in regular intervals whether there are new samples for analyzing. If the daemon is terminated, there is no more variant analysis.
Note: `PipelineDB.sh` currently must be started with an absolute pathname (`/var/pipeline/PipelineDB.sh` instead of `./PipelineDB.sh`)

9. Recommended configuration / Best practices

As AMLVaran was intended for clinical use in a specified setting, the process of uploading a sample on user site has been designed as easy-to-use as possible. When uploading a sample, only the target panel to be used for analysis and some information to identify the sample need to be specified.

The configuration of the variant calling pipeline and the selection of the variant calling tools are to be done by the system's administrator (with bioinformatics background) once when installing the software.

AMLVaran is shipped with a fully functional, pre-configured variant calling pipeline, which resembles the appreci8 algorithm. All tools and parameters are configured with reasonable default settings that have been carefully optimized and validated with various cancer datasets.

So, a recommended workflow is, to install the software as described in chapter 7, and start with the provided default settings that should work pretty well for most cancer scenarios.
The recommended settings are described in detail in the parameter's documentation at https://amlvaran.uni-muenster.de/doc/Version0.pdf.

For custom adaptations, we recommend to start with tuning the filter settings (in the Filtering area resp. on the appreci8 section in the AMLVaran web interface):

1.) select the thresholds for minimum coverage and allelic frequency, that your variants should fulfill
2.) adjust thresholds for allelic frequencies in reference exome sequencing projects (like 1000Genomes or ExAC), when a variant shall be classified as polymorphism
3.) disable some of the appreci8 scoring criteria, you do not agree with, or adjust their weightings

If you need special customizations, you may also remove some variant calling tools (e.g. with the highest runtime) or add your favorite variant calling tools to the pipeline, as described in chapter 10 of this document. For adaptation to a non-myeloid disease, you should modify the stored hotspot and driver mutations to be presented in the Quick overview and the rules for generating diagnostic recommendations, as described in chapter 11 of this document.

Although the default settings should produce good results for many cases, it is important that the analysis and filtering settings are validated together with the given lab parameters as an overall system, in order to get reliable variant calling results.
This validation has to be done by the system's administrator at the local facility.

10. How to add a variant caller?

By default, the following variant callers have been preconfigured:

a) Vardict: https://github.com/AstraZeneca-NGS/VarDict
b) LoFreq 2.1.2: http://csb5.github.io/lofreq/
c) GATK 3.3: https://software.broadinstitute.org/gatk/
d) samtools 1.3: http://www.htslib.org/
e) VarScan 2.3.9: http://dkoboldt.github.io/varscan/
f) freebayes 1.0.2: https://github.com/ekg/freebayes
g) SNVer 0.5.3: http://snver.sourceforge.net/
h) Platypus: http://www.well.ox.ac.uk/platypus

All caller tools to be used must be installed and configured. The configuration of the above tools is already included in our source code. In our docker image, these callers (with the exception of GATK due to licensing issues) are already pre-installed.

In order to configure a variant caller, a script `<Caller>.sh` must be created under `/pipeline/Callers`, which receives 2 command line arguments:

`$1` is the directory in which the sample to be analyzed is located, in the following `$dir`
`$2` is the name of the sample, in the following `$sample`.

The raw data can be accessed inside the script using `$dir/${sample}.bam`.

The script must file its output as `$dir/<Caller>/${sample}.vcf`

If the script outputs multiple files, they must all be located in the `$dir/<Caller>/` folder and have the filename extension `.vcf`.

Additionally a meta-file `<Caller>.fmt` must be created in the directory `/pipeline/Formats`, which specifies the output format of the caller. Some sample formats are already included. For more information on the specification of this format, please refer to the documentation of Variant Tools.

11. How to configure AMLVaran for another disease entity?

(AML)Varan can also be used for other disease entities.

All you need to do is adapt the curated data stored for hotspots and driver mutations.

This is an administrative process and not to be carried out by the end user, therefore not intended via the web interface. Accessing the database, however, makes it easy to store new information:

- First, a new evaluation "version" must be created.
- Then create a line in the table `tgt_KnownMutations` for each hotspot region, specifying its genomic coordinates. If the driver mutation can be located in more than one range, just add a line for each range and assign the same `MutationID` to it.
- The table `rul_Diagnosis` contains rule-based diagnostic recommendations that can be output on the basis of official guidelines. Each recommendation must have a unique `RuleID`.
- The `rul_Mutations` table specifies the conditions, under which a diagnostic advice is displayed. One or more `MutationIDs` from `tgt_KnownMutations` must be assigned to each diagnostic entry (`RuleID`). A condition can also include a combination of more than one mutation, and it can be specified, which ones have to be present and which ones have to be absent. The conditions can be linked by AND (each condition must be fulfilled), by adding one line per condition with the same `RuleID`. If a OR combination is to be resembled, just add another `RuleID` to the `rul_Diagnostics` and assign the same diagnostic text to it.

Finally, the new "version" can be displayed with all already processed or new samples by calling the URL `https://.../results.php?sid=x` with the addition `&version=y`.

The version displayed by default is saved for each sample in the `samples` table and can be changed there.
