# Supplementary material for: AMLVaran: a software approach to implement variant analysis of targeted NGS sequencing data in an oncological care setting
Source: BMC Med Genomics. 2020 Feb 4;13:17. doi: 10.1186/s12920-020-0668-3 (PMC7001226; doi:10.1186/s12920-020-0668-3)
Supplement: Supplementary file 3 — Additional file 3 The variant calling tools and parameters, as well as filter scheme used to generate the described results. [file 12920_2020_668_MOESM3_ESM.pdf]

Variant Calling Tools:

Caller	Version	Parameters
VarDict (Java)	1.5.5	**vardict**<br>-C (indicate chromosomes by numbers)<br>-f 0.01 (threshold for allele frequency)<br>-h (print a header row)<br>-c 1 (column for chromosome)<br>-S 2 (column for region start)<br>-E 3 (column for region end<br>-g 4 (column for gene name)
LoFreq	2.1.2	**lofreq** call<br>--call-indels
GATK*	3.5	**gatk –T BaseRecalibrator**<br>--maximum_cycle_value 1500<br>--covariates:<br>ContextCovariate, CycleCovariate, QualityScoreCovariate, ReadGroupCovariate<br>--knownSites:<br>dbSNP 138.b37.vcf, Mills_and_1000G_gold_standard.indels.b37.vcf, 1000G_phase1.indels.b37.vcf<br>-nct 1<br>**gatk –T PrintReads**<br>-BQSR<br>**gatk –T HaplotypeCaller**<br>--standard_min_confidence_threshold_for_calling 30.0<br>--standard_min_confidence_threshold_for_emitting 10.0<br>--downsample_to_coverage 1500<br>--max_alternate_alleles 9<br>--dbsnp dbsnp_129.b37.vcf<br>--num_cpu_threads_per_data_thread 1
samtools	1.3	**samtools** mpileup<br>--min-MQ 1<br>--BCF<br>--uncompressed<br>--output ${output}.bcf ${input}.bam<br>**bcftools** call<br>--variants-only<br>--multiallelic-caller<br>--output-type v (uncompressed vcf)<br>--output ${output}.vcf ${input}.bcf
VarScan	2.4.0	**samtools mpileup** --output ${output}.bcf<br>**varscan mpileup2snp** ${input}.bcf<br>**varscan mpileup2indel** ${input}.bcf
FreeBayes	1.0.2-6	**freebayes**<br>--min-alternate-fraction 0.01
SNVer	0.5.3	**snver**<br>-b 0.01 (discard locus with ratio of alt/ref below threshold)
Platypus	0.8.1	**platypus** callVariants<br>--filterDuplicates=0<br>--minFlank=0

* GATK is not delivered with the software due to license restrictions.

Annotation databases

Database	Version	Release date	Reference
**dbSNP**	v151	2017-10-06	https://www.ncbi.nlm.nih.gov/projects/SNP/
**ClinVar**<br>clinical variant database		2018-09-30	https://www.ncbi.nlm.nih.gov/clinvar/
**COSMIC***<br>Catalogue of Somatic Mutations in Cancer	v86	2018-08-01	https://cancer.sanger.ac.uk/cosmic
**1000 Genomes Project**<br>alternative allele frequency data for autosomes (ALL, EURopean)	Phase 3	2013-05-02	http://www.internationalgenome.org
**ExAC**<br>65000 exome allele frequency data for ALL and NFE (Non-finnish European)	v0.3	2015-11-29	http://exac.broadinstitute.org
**PROVEAN** scores (v1.1) on all possible single AA substitutions and deletions in human proteins from Ensembl 66	v1.1		http://provean.jcvi.org
**dbNSFP**<br>database of human non-synonymous SNPs and their functional predictions	v3.5	2017-08-06	Liu X, Jian X, and Boerwinkle E. 2011. dbNSFP: a lightweight database of human non-synonymous SNPs and their functional predictions. Human Mutation. 32:894-899.<br>Liu X, Wu C, Li C and Boerwinkle E. 2016. dbNSFP v3.0: A One-Stop Database of Functional Predictions and Annotations for Human Non-synonymous and Splice Site SNVs. Human Mutation. 37:235-241. [preprint]
Transcripts and Exons for GRCh37	Ensembl Release 75	Mar 2014	https://www.ensembl.org/index.html
**SNPeff**	v4.2		http://snpeff.sourceforge.net

* COSMIC is not delivered with the software due to license restrictions.

Basic filtering step

Filter out variants with…

- Low BaseQuality of alternative allele (threshold: 15)
  AND:
  (reads with reference allele are present) OR (BaseQuality of reference allele >15)

- BaseQuality of reference allele by 7 higher than of alternative allele

- Low BaseQuality of reference allele (≤ 15)
  AND high number of reads with reference allele (> 30)

Calculation of artifact / polymorphism score

Criterion	Arti	Poly
**Occurance in other samples**		
☑ No occurance in any other sample (NrSamples = 1)		-1
☑ (no Hotspot) AND (same variant in > 50% of all samples)	+2	
☑ Nr of samples with same variant > 3	+2	+1
☑ and 90 % of these samples have VAF > 0.85	+2	
**Allelic Frequency / Prediction**		
☑ NOT previous AND "unplausible" allelic frequency (< 0 OR between 0.35 - 0.65 OR > 0.85)		+1
☑ NOT previous AND Provean score ≥ -1.5	+1	
☑ Provean score ≥ -1.5		+1
☑ Provean score ≤ -4	-1	-1
☑ Variant Allelic Frequency (VAF) < 0.02	+2	
**Type of Variant**		
☑ "stop_gained" mutations (stop_gained suchen)		-1
☑ "inframe" mutations, but not "stop_gained"		+1
**Insertions / Deletions**		
Variant is an insertion / deletion / complex indel		
☑ Different variants at same locus found in other samples?	+1	
☑ VAF < 0.05	+1	
**StrandBias**		
Small StrandBias (p ≥ 0.001):		
☑ alternative on Forward strand ≤ 2 and reference ≥ 15	+1	
☑ alternative on Reverse strand ≤ 2 and reference ≥ 15	+1	
Large StrandBias (p < 0.001):	+1	
☑ alternative on both strands ≥ 10	-1	
☑ alternative on Forward strand ≤ 2 and reference < 15	-1	
☑ alternative on Reverse strand ≤ 2 and reference < 15	-1	
**Callers**		
☑ Variant found by only one caller	+1	
☑ Variant found by 4 callers	-1	
☑ Variant found by 5 callers	-2	
☑ Variant found by ≥ 6 callers	-3	+1
☑ Called by LoFreq and FreeBayes and VarDict	-3	

Databases

Databases to be used:

- [x] COSMIC *with not SNP and "haematopoietic and lymphoid tissue" > 20
- [x] ClinVar *with significance rating as "(likely) pathogenic"
- [x] dbSNP *with PM_flag or not in v129
- [x] dbSNP v129 (wird in NrAnyDBs zweifach gezaehlt!)
- [x] 1000Genomes, threshold: > 0.001
- [x] ESP6500, threshold: > 0.03
- [x] ExAC, threshold: > 0.000

Variant not present in any of the previous databases

- [x] and VAF < 0.1 — +1
- [x] and same variant in > 50% of all samples — +1
- [x] Variant matching thresholds in no (non-clinical*) database — -1
- [x] Variant matching thresholds in 2 or 3 (non-clinical*) databases — +1
- [x] Variant matching thresholds in ≥ 4 (non-clinical*) databases — +2
- [x] Variant found in ≥ 2 disease associated DBs* — -1
- [x] Variant identified as "Precious mutation" (PM) by dbSNP — -2

Known Hotspots

- [x] Variant in a known hotspot mutation site — -3
- [x] Not a known hotspot, but "Precious mutation" by dbSNP — -1

Finally: Exclude improbable polymorphisms

High Polymorphism score (≥ 2), no hotspot AND:

- [x] VAF ≤ 0.1 — +5
- [x] VAF ≤ 0.2 — +2
- [x] frameshift_mutation — +2

Classification

Artifact	Artifact score ≥ 0
likely Polymorphism	(no hotspot, no frameshift and high VAF) AND Polymorphism score ≥ 2
Polymorphism	(no hotspot, no frameshift and high VAF) AND Polymorphism score ≥ 3<br>OR<br>(Polymorphism score ≥ 2) AND (Cosmic NrHaemato ≤ 100)
Probably True	None of the above
